# G-quadruplex forming sequences in the genome of all known human viruses: A comprehensive guide

**DOI:** 10.1371/journal.pcbi.1006675

**Published:** 2018-12-13

**Authors:** Enrico Lavezzo, Michele Berselli, Ilaria Frasson, Rosalba Perrone, Giorgio Palù, Alessandra R. Brazzale, Sara N. Richter, Stefano Toppo

**Affiliations:** 1 Department of Molecular Medicine, University of Padova, Padova, Italy; 2 Department of Statistical Sciences, University of Padova, Padova, Italy; Masarykova Univerzita, CZECH REPUBLIC

## Abstract

G-quadruplexes are non-canonical nucleic-acid structures that control transcription, replication, and recombination in organisms. G-quadruplexes are present in eukaryotes, prokaryotes, and viruses. In the latter, mounting evidence indicates their key biological activity. Since data on viruses are scattered, we here present a comprehensive analysis of potential quadruplex-forming sequences (PQS) in the genome of all known viruses that can infect humans. We show that occurrence and location of PQSs are features characteristic of each virus class and family. Our statistical analysis proves that their presence within the viral genome is orderly arranged, as indicated by the possibility to correctly assign up to two-thirds of viruses to their exact class based on the PQS classification. For each virus we provide: i) the list of all PQS present in the genome (positive and negative strands), ii) their position in the viral genome, iii) the degree of conservation among strains of each PQS in its genome context, iv) the statistical significance of PQS abundance. This information is accessible from a database to allow the easy navigation of the results: http://www.medcomp.medicina.unipd.it/main_site/doku.php?id=g4virus. The availability of these data will greatly expedite research on G-quadruplex in viruses, with the possibility to accelerate finding therapeutic opportunities to numerous and some fearsome human diseases.

## Author summary

G-quadruplexes are nucleic acid non-canonical structures that have been implicated in the regulation of different biological processes of many organisms. Their presence has been demonstrated also in several viral pathogens, providing new insights into viruses’ biology and potentially serving as drug targets. Although experimental validation is needed to confirm the actual folding of G-quadruplexes, they can be inferred *in silico* directly from the nucleotide sequence. Several computational methods exist for this purpose, but they are all limited to the analysis of independent sequences. Since viral genomes can be highly variable, G-quadruplexes with important functional roles are expected to be conserved among strains and isolates belonging to the same viral species. Here we aimed at characterizing the potential quadruplex-forming sequences (PQS) content in the genome of viral human pathogens and assess their degree of conservation in each viral species. We demonstrate that many viruses possess more PQSs than expected from their nucleotide composition and some of them are highly conserved within single viral species, claiming some biological roles. We provide a website where the results of our analyses are displayed for each virus with interactive graphics. This work is intended as a resource that can guide scientists in the choice of the most promising candidates for functional characterization.

## Introduction

G-quadruplexes (G4s) are nucleic-acid secondary structures that may form in single-stranded DNA and RNA G-rich sequences under physiological conditions [1]. Four Gs bind via Hoogsteen-type base-pairing to yield G-quartets: stacking of at least two G-quartets leads to G4 formation, through π-π interactions between aromatic systems of G-quartets. K^+^ cations in the central cavity relieve repulsion among oxygen atoms and specifically support G4 formation and stability [2]. In the human genome, potential quadruplex-forming sequences (PQS) are clustered at definite genomic regions, such as telomeres, oncogene promoters, immunoglobulin switch regions, DNA replication origins and recombination sites [3]. In RNA, G4s and PQSs were mapped in mRNAs and in non-coding RNAs (ncRNAs) [4], such as long non-coding RNAs (lncRNAs) [5] and precursor microRNAs (pre-miRNAs) [6] indicating the potential of RNA G4s to regulate both pre- and post-transcriptional gene expression [7, 8].

Viruses are intracellular parasites that replicate by exploiting the cell replication and protein synthesis machineries. Viruses that infect humans are very diverse and, according to the Baltimore classification, they can be divided in seven groups based on the type of their genome and mechanism of genome replication: DNA viruses with 1) double-stranded (ds) and 2) single-stranded (ss) genome; RNA viruses with 3) ds genome, or ss genome with 4) positive (ssRNA (+)) or 5) negative (ssRNA (-)) polarity; 6) RNA or 7) DNA viruses with reverse transcription (RT) ability, whose genome is converted from RNA to DNA during the virus replication cycle (Table 1). Each of these classes possesses a peculiar replication cycle [9].

**Table 1:** Virus families.

Genome nature	DNA		RNA			DNA and RNA	
Group	1	2	3	4	5	6	7
Genome type	dsDNA	ssDNA	dsRNA	ssRNA (+)	ssRNA (-)	ssRNA (RT)	dsDNA (RT)
**Virus family**	Herpes	Anello	Reo	Corona	Rhabdo	Retro	Hepadna
	Adeno	Parvo		Astro	Filo		
	Papilloma			Calici	Paramyxo		
	Polyoma			Flavi	Arena		
	Pox			Picorna	Bunya		
				Toga	Orthomyxo		

Virus families divided according to their genome and mechanism of replication. The suffix word “viridae” for each virus family has been omitted.

The presence of G4s in viruses and their involvement in virus key steps is increasingly evident in most of the Baltimore groups [10, 11]. In the dsDNA group, G4s were described in both *Herpesviridae* and *Papillomaviridae* families [12–20]. In ssDNA viruses, the presence of G4s was reported in the adeno-associated virus genome [21]. RNA G4s were described in the genomes of both ssRNA (+) (i.e. Zika, hepatitis C virus (HCV) [22, 23], and the severe acute respiratory syndrome (SARS) coronavirus [24, 25]) and ssRNA (-) viruses (i.e. Ebola virus [26]). A G4 was also detected in hepatitis B virus (HBV) genome, the only member of dsDNA viruses with RT activity [27]. Finally, functionally significant G4s were identified both in the RNA and DNA proviral genome of the human immunodeficiency virus (HIV), a retrovirus belonging to group 6 (Table 1) [28–35], and [33, 34]in the LTR region of lentiviruses in general (ssRNA RT) [36].

**Fig 1:**
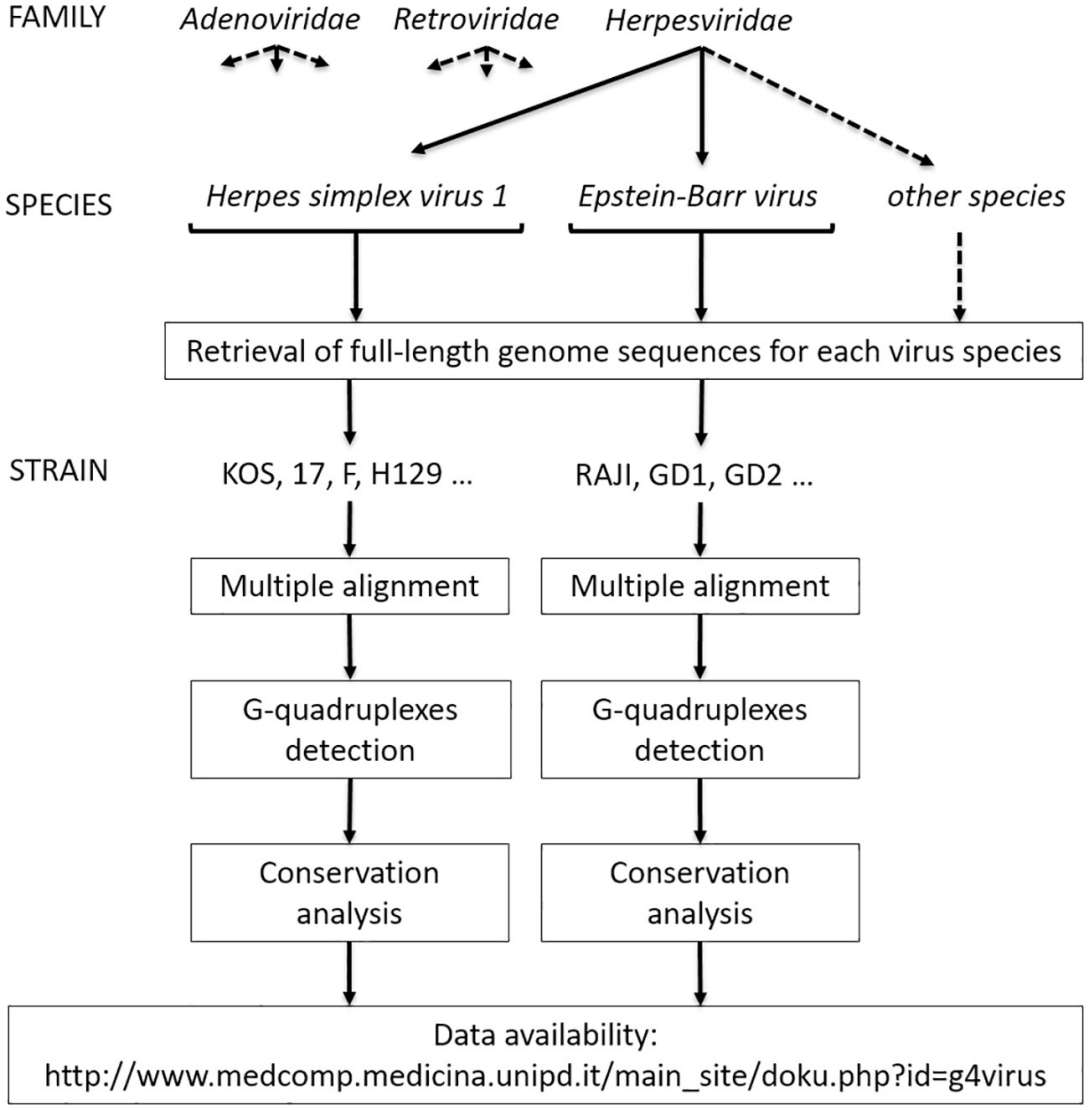
Example of virus classification in families, species and strains with data analysis flowchart. The conservation analysis was performed among the strains within each virus species.

Given this amount of scattered data, we here aimed at analyzing the presence of PQSs in the genome of all known viruses that can cause infections in humans. The analysis is performed at two distinct levels, globally for each viral genome and individually for each detected PQS. We asked the following: is the number of PQSs found in a viral genome simply due to chance, hence trivially reflecting genomic G/C content? And how much is each PQS conserved among the strains belonging to a viral species? To address these questions, we collected the whole viral genomes deposited in databanks, scanned them to detect all PQSs, and performed different statistical evaluations following the data analysis workflow shown in Fig 1. The detailed information on PQSs present in each human virus is available in an easily accessible web site with interactive graphics and genome browser visualization tools (http://www.medcomp.medicina.unipd.it/main_site/doku.php?id=g4virus).

## Results

### Detection of G4 patterns in all known human viruses

All known viruses that cause infections in humans, according to the Viral Zone ExPASy web site (http://viralzone.expasy.org/all_by_species/678.html), were grouped in 7 classes according to Baltimore classification, which takes into account the viral genome nature: dsDNA, ssDNA, dsRNA, ssRNA(+), ssRNA(-), ssRNA(RT) and dsDNA(RT). Different replication strategies and structural similarities allow to further divide viruses in families (Table 1). The complete list of reference sequences for each virus included in the analyses is reported in S1 Table.

PQSs in viral genomes were searched by looking for the following patterns: [G(2)N(1–7)](3)G(2), [G(3)N(1–12)](3)G(3) and [G(4)N(1–12)](3)G(4), where both island and loop lengths were chosen to provide a comprehensive detection. We decided to expand the search to PQSs with very short islands and quite extended loops for the following reasons: first, the folding of PQS with GG-islands has been previously demonstrated in viruses [32]; second, since many viruses possess a RNA genome, and considering that RNA G4s are more stable than their DNA counterparts [37], PQSs with only two tetrads have a reasonable chance to fold in viral RNA genomes or in their intermediates. Finally, while long loops are known to destabilize G4 structures, their presence is anyway compatible with the folding of stable G4s at physiological temperature [38]. PQSs with bulged islands [39] and intermolecular G4s are not considered in the present study.

**Fig 2:**
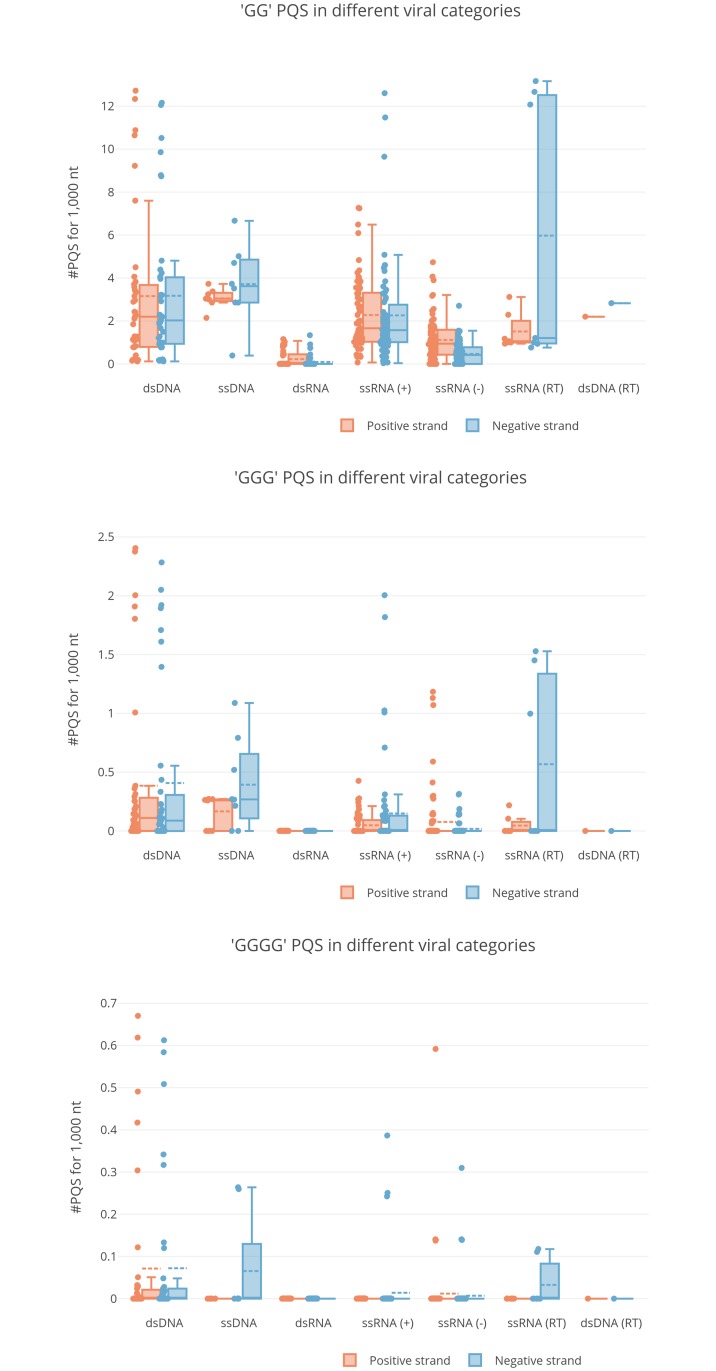
Box and whisker plots of PQSs in different virus classes. Each panel refers to the indicated type of G-island (GG, GGG, GGGG). The abundance of PQSs per 1 kb of viral genome is reported in the y-axis (for each viral species, the median value among all available strains is used) and the different virus categories in the x-axis. Boxplots are delimited by the first and third quartile and the straight and dotted lines drawn inside are the median and mean values, respectively, of the PQS distribution. The single observations are reported as dots close to the box plot. Whiskers delimit all the points that fall above/below the third/first quartile plus/minus 1.5 times the interquartile range (IQR). Orange and blue box plots refer to positive and negative strand respectively.

PQSs were searched in the positive and negative strand of each virus genome sequence, since both filaments are present and important in different stages of the viral replicative cycle of all virus classes. As the length of virus genomes greatly varies, i.e. from 235,646 nucleotides (nts) of the human cytomegalovirus (HCMV) to 1,682 nts of hepatitis delta virus (HDV), we reported the number of PQS independently of the genome length by normalizing their number per 1,000 nts (Fig 2). The PQS distribution for both the positive and negative strands is shown as a box plot for each Baltimore virus class, whereas the PQS count for each virus within each class is shown as a dot besides the box plot (Fig 2). The negative strand of retroviruses (ssRNA (RT) viruses), ssDNA viruses and both strands of dsDNA viruses showed the largest presence of PQSs made of GG-, GGG- and GGGG-islands (box plots, Fig 2). Both strands of genomes of single virus families belonging to these groups and to ssRNA (+) and ssRNA (-) were enriched in PQSs of all G-islands types (dot plots, Fig 2). Conversely, dsRNA and dsDNA (RT) viruses notably lacked the presence of PQSs.

**Fig 3:**
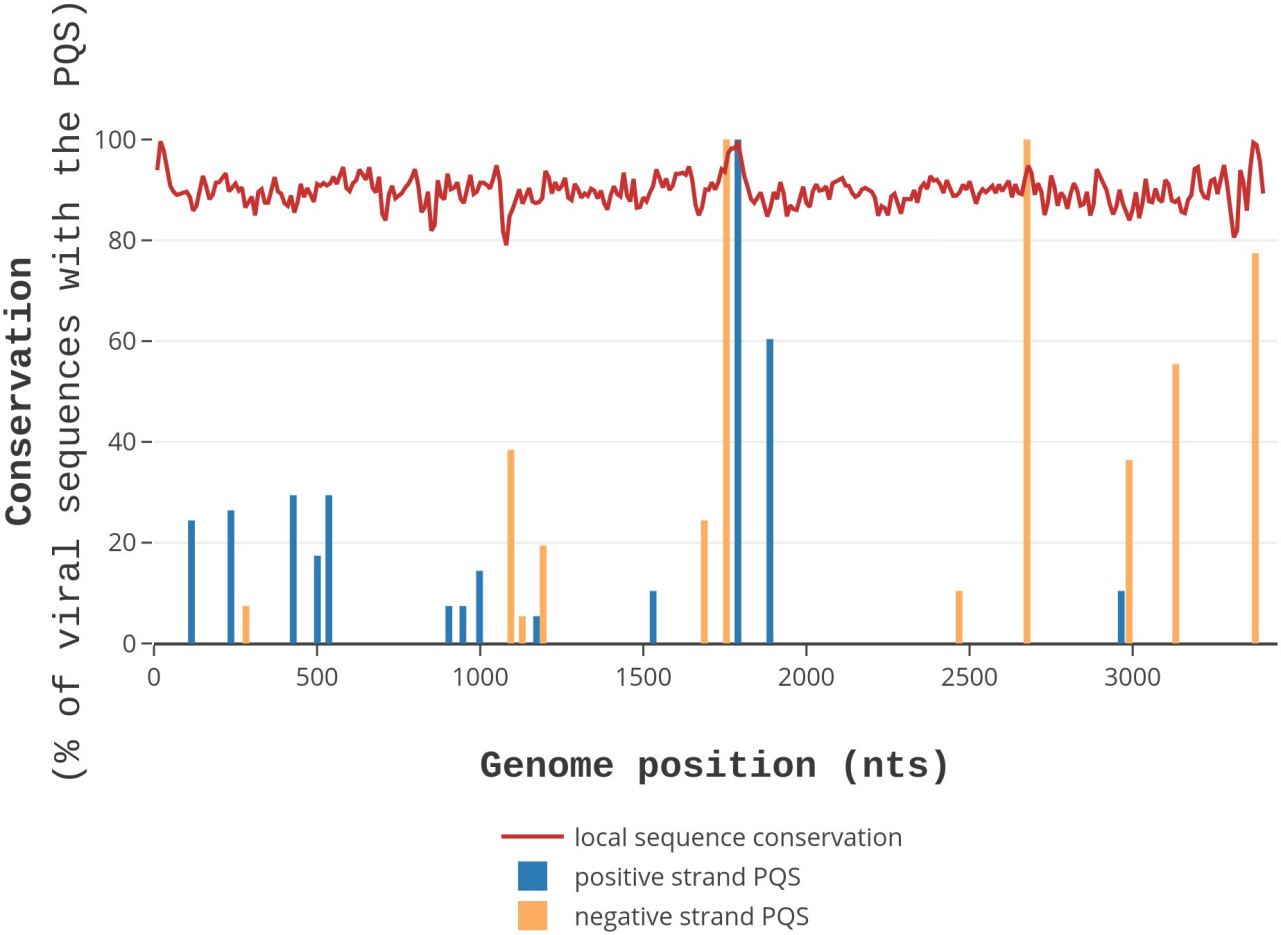
Conservation of PQSs and viral genomes. PQSs formed by GG-islands in the S segment of lymphocytic choriomeningitis virus are shown. PQSs found in the positive and negative strands are indicated as blue and orange vertical bars, respectively, while the height of bars represents the G4_SCI (the conservation of G-islands). Local sequence conservation (LSC) of viral genomes is shown as a red broken line. The x-axis indicates genome position, the y-axis the conservation %.

Then, we evaluated the conservation of PQSs among different strains of each viral species, hypothesizing that the presence of a conserved PQS within a less conserved genome environment could be an indication of a G4 with a biological function [40]. To allow for the evaluation of PQS conservation in the local context of viral genomes, we computed the “G4 scaffold conservation index” (G4_SCI) for each PQS in each virus species. This value measures the degree of conservation of G-islands that are necessary and sufficient to form a PQS: the higher the score, the higher the conservation of the PQS. An example of the results from such analysis is reported in Fig 3 for the lymphocytic choriomeningitis virus (segment S): all PQSs detected in the virus are plotted as vertical bars, the height and position of which represent the G4_SCI on the y-axis and the genome coordinates on the x-axis, respectively. In addition, the local sequence conservation (LSC) of the viral genome, calculated with a sliding window approach on all available viral sequences, is reported alongside as a red broken line. This visualization method allows the prompt identification of the presence, position, and conservation of G-islands within PQSs, together with the overall local conservation of the genomic context. Moreover, the degree of conservation of the connecting regions (loops) with respect to G-islands (the *loop_conservation* value) was calculated as the difference between G4_SCI and LSC. Positive and negative *loop_conservation* scores indicate, respectively, lower and higher conservation of connecting regions compared to the conservation of G-islands. Values close to zero mean that both G-islands and connecting loops show the same level of sequence conservation. In Fig 3, three PQSs formed by highly conserved GG-islands are shown for the S segment of lymphocytic choriomeningitis virus, present in genomic regions both well and less well conserved (Fig 3 at positions 1,790 in the positive strand, 1,760 and 2,680 in the negative strand). This kind of analysis is available for all PQSs of all human virus species at http://www.medcomp.medicina.unipd.it/main_site/doku.php?id=g4virus (*loop_conservation* values are included in tarballs downloadable for each viral class of the Baltimore classification, whereas each virus species has a dedicated page displaying all graphical representations).

To assess the results, we retrieved from the literature all the available experimentally validated G4s detected in human viruses. All patterns were confirmed also by our analysis and the complete list is reported in S2 Table, together with the genomic coordinates of the predicted PQSs.

### Statistical evidence of the presence of PQSs in the human virus genomes

G4 formation may be largely affected by G/C content, which greatly varies in viral genomes (from 76% of Cercopithecine 2 herpes virus to 27% of Yaba like disease virus). Moreover, it has been shown that some di- and trinucleotides are over- or under-represented in certain viruses [41, 42] and, in the context of PQSs, this means that their abundance could be biased by unexpected frequencies of guanine homopolymers. G-island frequencies higher or lower than expected would lead to a potential over- or under-representation of PQSs, respectively.

To check whether the presence of PQSs was statistically relevant or whether it occurred by pure chance, we compared the results obtained from real viral genomes with those obtained by two different simulation strategies. The first one (single nucleotide assembling) assumes that the occurrence of each DNA base in the genome is independent [43]; the second (G-island reshuffling) considers that short sequences of a given length (k-mer) could be over- or under-represented in certain viral genomes [41, 42]. In the former case, sequences were generated with the same composition of nucleotides but different order with respect to references; in the latter, sequences were produced by reshuffling the positions of G-islands while keeping constant their number.

For each virus and simulation strategy, we produced 10,000 random sequences, which were screened with our PQSs detection pipeline. Real and simulated data were compared by computing a P-value, defined as twice the smaller proportion of simulated sequences that exhibit, respectively, a higher and lower count of PQSs as compared to the median value of all the available complete genome sequences for a certain virus. Hence, a P-value close to 1 means that the median PQS content in real viral sequences is not significant if compared to a random distribution; conversely, a P-value close to 0 means that PQS content is highly significant. This interpretation holds independently of the length of the genome and/or of the prevalence of either G/C bases or G-islands, as we compare the number of PQSs in a viral genome with the one we would expect in a simulated genome of the same length and of either the same base or G-island composition. To account for possible high discreteness of the data, a less conservative version of the P-value, called the mid-P value [44], was used. Segment diagrams of the mid-P values of the Baltimore grouped viruses are reported in S1 and S2 Figs [45]. The number of viruses whose median PQS count is significant at the 10% level is listed in Table 2 (virus names in S3 Table) with the indication of whether this median count is either higher or lower than the PQS count in simulated sequences.

**Table 2:** Relative abundance of viruses having a PQS content significantly different between real and simulated viral genomes.

	Number of viruses significantly different vs. randomization at single nucleotide level		Number of viruses significantly different vs. randomization at the G-island level		Number of viruses significantly different vs. both randomization	
G-island pattern	PQS more abundant in real sequences	PQS more abundant in simulated sequences	PQS more abundant in real sequences	PQS more abundant in simulated sequences	PQS more abundant in real sequences	PQS more abundant in simulated sequences
**GG (positive strand)**	83/218 (38.1%)	9/218 (4.1%)	52/217 (24.0%)	4/217 (1.8%)	49/217 (22.6%)	3/217 (1.4%)
**GG (negative strand)**	83/187 (44.4%)	2/187 (1.1%)	67/187 (35.8%)	1/187 (0.5%)	59/186 (31.7%)	0 (0%)
**GGG (positive strand)**	41/78 (52.6%)	3/78 (3.8%)	40/75 (53.3%)	0 (0%)	34/74 (45.9%)	0 (0%)
**GGG (negative strand)**	32/68 (47.1%)	3/68 (4.4%)	32/69 (46.4%)	0 (0%)	28/68 (41.2%)	0 (0%)
**GGGG (positive strand)**	17/19 (89.5%)	0 (0%)	17/17 (100%)	0 (0%)	17/17 (100%)	0 (0%)
**GGGG (negative strand)**	23/28 (82.1%)	0 (0%)	23/25 (92.0%)	0 (0%)	23/25 (92.0%)	0 (0%)

The number of viruses where the amount of PQSs is significantly different at 10% level between real and simulated sequences is reported (with percentages in brackets). Values and percentages were calculated considering only viruses containing at least one PQS either in real or simulated sequences (this explains differences in denominators). The table reports significant values for either one of the two simulations (randomization of viral genomes at single nucleotide or at G-island levels) or both.

Our data show that most members of the dsDNA, ssDNA, and ssRNA (RT) present a highly significant content of PQSs formed by GG-, GGG- and/or GGGG-islands in one or both strands. ssRNA (-) and ssRNA (+) classes are heterogeneous since some viruses are highly significant in any PQS category (from GG- to GGGG-islands), while others are not (see below). The presence of PQSs in members of the dsRNA group is notably less significant. Interestingly, few viruses display a smaller amount of PQSs than expected: both *Sagiyama virus* and *Human coronavirus HKU1* are depleted of PQSs belonging to GG-islands category in the positive genome strand when compared with both simulation strategies based on single nucleotide assembling and GG-island reshuffling. In addition, *Human parainfluenza virus 2* is poor of PQSs made of GG-island in the positive genome strand but is enriched in both GG- and GGG-type PQSs in the negative strand.

Overall, if we consider the viruses that contain at least one PQS in either the real or the simulated genomes, we observe that the increase in G-islands’ length corresponds to a decrease in the absolute number of viruses containing PQSs, but it also corresponds to a dramatic increase in the fraction of them that is statistically significant.

By looking at the family level of viral classification, which is far more homogeneous than the Baltimore groups, some virus families emerge as prominently enriched in PQSs. Among them, *Herpesviridae* is not only the one with the highest PQS content, but most of its members display significantly more PQSs than expected in both genome strands and in all considered G-island lengths. Notably, some of the viruses belonging to *Herpesviridae* and showing the highest G/C content are statistically enriched in PQSs. This suggests that simply having a high G/C content is not a sufficient condition to justify the presence of such a high number of PQSs. Other viral families that are consistently enriched in PQSs are *Adenoviridae* and *Papillomaviridae*, especially in GG- (both strands) and GGG-island (positive strand) types. *Poxviridae* and *Parvoviridae* show an enrichment of GG-type PQSs in both genome strands, whereas the same pattern is enriched in the positive strand of all *Anelloviridae* members and in the negative strand of most *Paramyxoviridae* and *Retroviridae* viruses. All other families are generally not enriched in PQSs in any of the evaluated categories, with only a few exceptions that are listed in the following: L segments of *Lassa virus* and *Lymphocytic choriomeningitis virus* (*Arenaviridae*), *Wu* and *Merkel cell polyomaviruses* (*Polyomaviridae*), *Salivirus* (*Picornaviridae*), M and S segments of respectively *Crimean-Congo hemorrhagic fever virus* and *Rift Valley fever virus* (*Bunyaviridae*).

**Fig 4:**
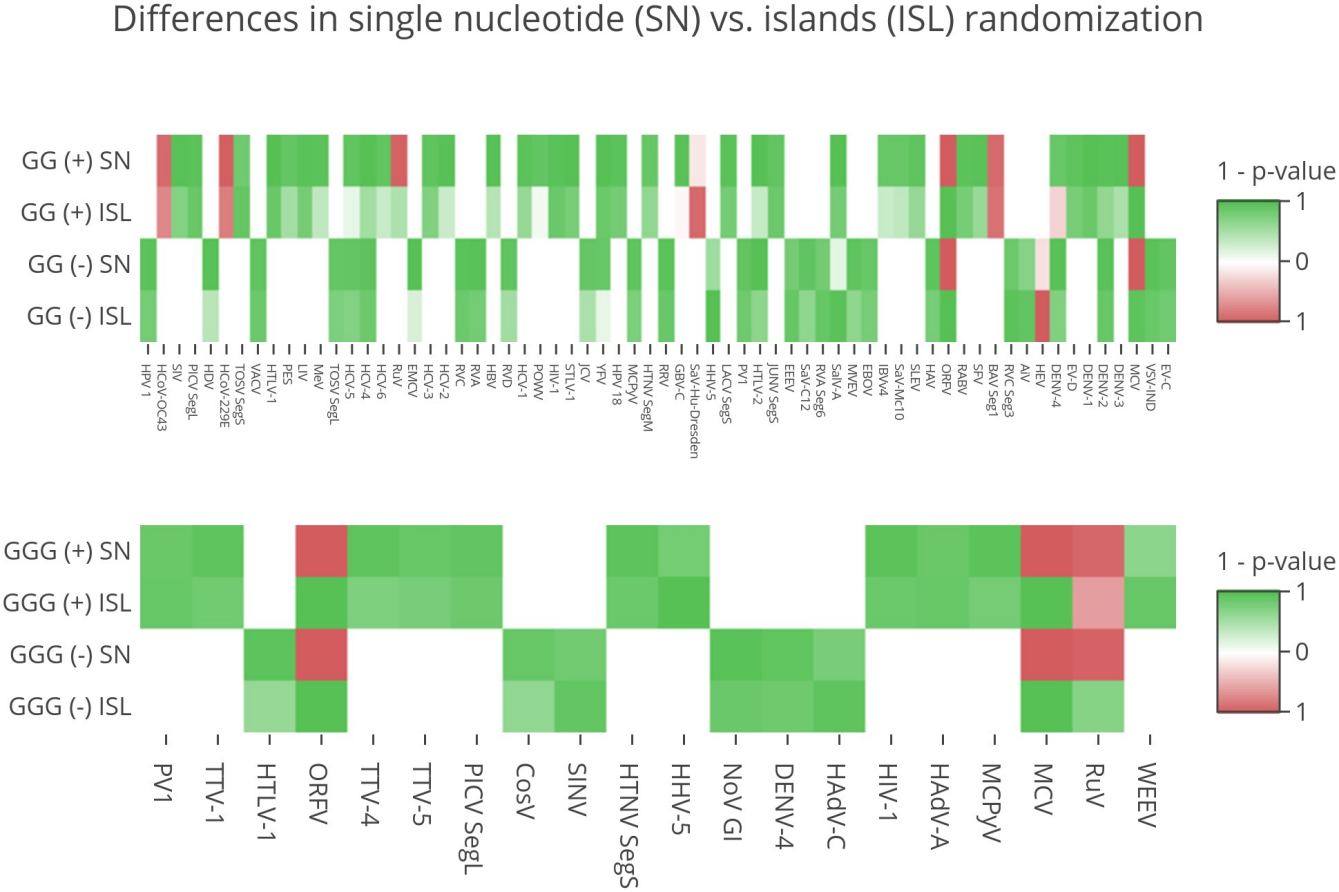
Different results from single nucleotide (SN) and islands (ISL) reshuffling strategies. Heatmaps show all the viruses which are significant in only one of the two simulations or that obtain discordant results. Green and red boxes indicate that PQSs are more abundant in real and simulated genomes, respectively, with color intensity proportional to p-value size.

By comparing the results obtained independently from the two simulation strategies it is possible to draw additional conclusions. First, in most cases the results are concordant, meaning that both simulations show similar trends in the statistical significance. Nonetheless, the overall number of viruses whose PQS content is significantly different with respect to simulated data is higher when real viral genomes are compared to those generated by single nucleotide assembling. This difference indicates that viral genome k-mer composition is indeed affecting the probability of randomly finding PQSs, at least in a proportion of viruses as shown in Fig 4: in the heatmaps, viruses that are significant in only one of the two simulations are reported for GG- and GGG-island patterns, whereas no such cases were found for GGGG-type PQSs.

Finally, some remarkable exceptions exist where both simulations return a significant p-value, but with an opposite meaning. This is the case of two members of the *Poxviridae* family, namely *Molluscum contagiosum virus* and *Orf virus*, which are enriched in GG- and GGG-type PQSs in both strands of their genomes if compared with the islands reshuffling simulation but show the opposite behavior when compared with the single nucleotide assembling (they are also reported in Fig 4). While the full meaning of this observation is not clear to us, it seems that these viruses possess far less PQSs than they could have, but at the same time they are able to cluster their relatively few G-islands in more PQSs than expected.

### Human virus PQSs position and overlap with genomic features

To check the prevalent positions of PQSs in virus genomes, we compared the coordinates of predicted PQSs with the available information regarding viral genome features. Genome coordinates were extracted for coding sequences (CDS), repeat regions (RR), 5’- and 3’-untranslated (UTR), and promoter regions. While CDS and RR are explicitly defined in RefSeq and GenBank databases, the annotation of UTRs and promoters is more inconsistent, being defined only for some viral species. For this reason, the annotations of genes and CDSs were exploited to indirectly extract the coordinates of 5’–and 3’–regulatory regions (see Materials and methods for details). To determine the localization of PQSs in viral genomes, the overlap extent between PQSs and genomic features was computed. Given the vast heterogeneity of the annotations reported in the feature fields, a manual revision was required to fix potential inconsistencies in annotations, regarding both keywords and coordinates. A revision was performed when possible, while controversial and uncertain annotations were not considered. These analyses are presented as bar charts for individual viral classes and G-island pattern types (GG-, GGG-, GGGG-island) (S3–S5 Figs). As regards the GGG-island type, the herpesvirus family of dsDNA viruses presents PQSs distributed along all the four identified genomic features, with a particularly high concentration in RR and, in some members, in the 5’–regulatory region. This feature is consistent with the reported extent of G4s in HSV-1, which are mainly clustered in the RR of the virus genome [12, 13]. Conversely, viruses belonging to the ssRNA (+) and ssRNA (-) classes show PQSs mainly grouped in CDS and in the 3’- and 5’-regulatory regions, respectively. HIV-1, belonging to ssRNA (RT) virus class, presents PQSs of the GGG-island type mainly in the RR and 3’-regulatory regions and in part in CDS. This distribution confirms previous data [28, 32]. Conversely, other retroviruses (ssRNA (RT)) such as HTLV-1 and HTLV-2, display PQSs in the CDS. Given the lower stringency of PQSs of the GG-island type, these are more widely distributed along the four identified genomic features, whereas the most stringent PQSs of the GGGG type, present only in herpesviruses (dsDNA) and HTLV-1 (ssRNA (RT)), show a clear-cut localization in the RR and CDS, respectively. These data indicate that the localization of PQSs in the viral genomes differs in virus classes.

### The number and type of PQSs are characteristic of virus classes

In this line of thinking, we asked whether the observed number of PQSs, and more precisely its statistical significance with respect to the two random assembling scenarios, is representative for a specific Baltimore class. To answer this question, we checked whether it is possible to classify each virus to one of the six classes considered, that is, dsDNA, ssDNA, dsRNA, ssRNA (+), ssRNA (-) and ssRNA (RT), based on the information of how significant its median PQS counts are. We used a classifier built on multinomial logistic regression, as this method is both interpretable and robust to unbalanced group sizes as long as the group sizes are large enough. To avoid the latter drawback, we excluded from the model fit the hepatitis B virus, the only virus classified as dsDNA (RT), and the two unclassified hepatitis delta and hepatitis E viruses. Six features were used to classify the viruses, i.e. the six mid-P values (those calculated for GG-, GGG-, GGGG-, both in the positive and negative strand) which qualify the PQS content of the real viral sequences. The values were multiplied by 1 or -1 depending on whether the median PQS count was over- or under-represented. Since real and corresponding simulated sequences contain the same base or G-islands composition, the classification model based on PQS content does not depend on the highly variable genome length and G/C content in the different virus classes but is specifically designed on the peculiar presence or absence of PQSs in each viral class. Furthermore, 34 viruses with no PQS count in all three G-island types in both the positive and negative strand and non-significant mid-P values at the 10% level were excluded from the analysis. We re-classified every viral genome used in our assessment using the discriminant function obtained from a leave-one-out analysis. This latter technique allowed us to accurately estimate how our classifier performs without the need to split our data into a training and a test set. The corresponding confusion matrix is given in Table 3 from where it is possible to extract the overall percentage of correct classifications that amount to 66.7% for the single nucleotide assembling model and 68.1% for the G-island reshuffling model. The agreement is good for the dsDNA, ssDNA, dsRNA, ssRNA (+) and ssRNA (-) classes. The two unclassified genomes of the hepatitis delta and hepatitis E viruses were classified as ssRNA (+).

**Table 3:** Confusion matrix for the semi-parametric classifier for G4 structure.

**Single nucleotide assembling model**		**Predicted class**						
		**dsDNA**	**ssDNA**	**dsRNA**	**ssRNA (+)**	**ssRNA (-)**	**ssRNA (RT)**	**Unclassified**
**True class**	**dsDNA**	33	0	0	1	4	1	0
	**ssDNA**	2	0	0	5	0	1	0
	**dsRNA**	0	0	13	0	5	0	0
	**ssRNA (+)**	4	0	0	45	13	0	0
	**ssRNA (-)**	3	0	8	18	48	0	0
	**ssRNA (RT)**	4	0	0	0	0	3	0
	**Unclassified**	0	0	0	2	0	0	0
**G-island reshuffling model**		**Predicted class**						
		**dsDNA**	**ssDNA**	**dsRNA**	**ssRNA (+)**	**ssRNA (-)**	**ssRNA (RT)**	**Unclassified**
**True class**	**dsDNA**	31	0	0	5	1	2	0
	**ssDNA**	4	0	0	2	2	0	0
	**dsRNA**	0	0	14	0	4	0	0
	**ssRNA (+)**	3	1	0	48	10	0	0
	**ssRNA (-)**	7	1	4	13	52	0	0
	**ssRNA (RT)**	3	0	0	3	1	0	0
	**Unclassified**	0	0	0	2	0	0	0

The number of viruses classified into the six Baltimore groups is shown, based on the two different simulation scenarios (single-nucleotide and G-island reshuffling). The classifier is based on a multinomial model and uses the one-sided mid-P values as features in combination with the information on whether the median PQS count is under- or over-represented.

## Discussion

In this work we provide: i) the list of PQSs present in all human virus genomes (both positive and negative strands), ii) their position in the viral genome, iii) the degree of conservation of both G-islands and loops vs. the genome, iv) the statistical significance of PQS abundance in each virus. Our data show that viruses belonging to dsDNA, ssDNA, ssRNA (RT) and, to a less extent, ssRNA (+) and ssRNA (-) display the largest presence of GG-, GGG- and GGGG-type PQSs (box plots, Fig 2) and that the presence of PQSs in all these virus groups is statistically significant (segment diagrams, S1 and S2 Figs). Both results support a role of G4s in the virus biology: indeed, some G4s predicted in this work were already reported in viruses and were shown to possess specific and different functions.

We evidenced some general trends and exceptions that are worth noting if seen in comparative terms among all viruses considered in this study. Starting from the general features we noted: i) high G/C content is not sufficient per se to generate a high number of PQSs, as observed in G/C rich members of *Herpesviridae* family that are richer of PQSs than expected. ii) The statistical significance of PQSs found in real sequences tends to decrease when G-islands reshuffling (ISL) is compared with the corresponding single nucleotide assembling (SN), as is appreciable from the heatmap in Fig 4 (more intense color in the heatmap boxes). This suggests that short sequences of a given length (k-mer) could be over- or under-represented in certain viral genomes, as already reported in the literature [41, 42]. We observed that viral genomes enriched in PQSs also contain a higher number of G-islands than expected from mere nucleotide composition, especially evident in the GG-islands. iii) The unevenly distribution of PQSs can be used to classify membership of a virus in its corresponding category. This was not predictable *a priori* but up to two-thirds of unequivocal assignments suggest that for some viruses the PQS content works as a distinctive feature. iv) PQS localization shows differences in some virus classes, but this outcome is still incomplete due to lack of information in the databases about virus genomic features and partitioning into regulatory and coding regions.

Some other interesting observations are worth reporting as either special cases or exceptions. To start with, the ssRNA (-) group is the most heterogeneous one, since some viral species are significantly enriched in PQSs up to the most extended G-island type (GGGG), while others lack this feature. Surprisingly, two viruses of the dsDNA group, which was generally highly enriched in PQSs, show a significantly lower presence of PQSs than expected in a random sequence with the same G/C content (SN, S3 Table), even though the opposite result was observed vs. simulated genomes with the same G-islands content as the real ones (ISL, S3 Table). These two viruses, i.e. *Molluscum contagiosum virus* and *Orf virus*, are the only ones belonging to genera other than the *Orthopoxvirus* within the *Poxviridae* family that cause skin lesions. Finally, dsRNA and dsDNA (RT) viruses are notably poor in PQSs and with mostly null statistical significance; however, single PQSs are highly conserved (e.g. rotavirus a segment 6), therefore still conveying potential biological interest.

These data indicate that PQSs are mainly present in ss-genome viruses, which in principle are more suitable to fold into G4s since they do not require unfolding from a fully complementary strand. The major exception to this evidence is the *Herpesviridae* family of dsDNA viruses. In this case, most PQSs reported here and also previously described [12, 13] form in repeated regions. It is possible that repeated sequences are more prone to alternative folding, as shown by several non-canonical structures that form in repeated regions of the DNA [46–49]. However, for some herpesviruses many PQSs are also present in regulatory regions, which may indicate yet undiscovered functional roles. To note that the investigation of PQSs was performed on a maximum window of 52 nucleotides in the case of isolated G4s. Alternatively, when more than four G-islands are found complying with the maximum distance allowed between consecutive islands, this window is extended as long as the rules are satisfied, thus including multiple distinct PQSs or potential isoforms. However, it is possible, especially for the ss genomes, that bases more distant in the primary sequence interact among each other, therefore expanding the repertoire of G4 structures.

The significant enrichment of PQSs in many viruses with respect to the corresponding randomized genomes is an indication that the clustering of G-islands did not occur by pure chance, suggesting a specific biological role of the G4 structures [40]. Complementary to this, the analysis of the PQS conservation highlights every PQS that is conserved among viral strains. Since one of the peculiarities of viral genomes is their fast mutation rate, the strong conservation of a specific G-island pattern among strains is per se an indication of the biological relevance of a PQS. In light of this, single conserved PQSs in viruses that do not display statistically significant PQS enrichment may retain key functional roles. The meaning of PQS conservation can have different explanations for the different viruses analyzed in this study. Given the high variability in the number of full-genome sequences available for each species, a more general evaluation of PQS conservation at higher taxonomic ranks (e.g. at the family level) could have been more informative. Nonetheless, generating and analyzing whole-genome multiple alignments involving different viral species, even if belonging to the same family, is almost prohibitive given the huge variability that is usually present in their genome sequences. Hence, the conservation of each PQS has to be considered on a case by case basis, exploiting the visualization tools provided in the website. As an example, an interesting approach could be looking at the discrepancy between the conservation of G-islands and connecting loops (*loop_conservation*) as an additional indication on the likeliness of biological implications of a specific PQS. A positive *loop_conservation* value highlights G-islands more conserved than their connecting loops, suggesting that only the PQS scaffold is required for mechanisms that are important for the virus life cycle, while the loops are dispensable. Considering the high mutation rate of viruses, this type of conservation indicates sequences where G4 formation is most likely essential. Equally conserved G-islands and loops (*loop_conservation* value = 0) imply that both the PQS scaffold and connecting loops are potentially relevant for the virus and probably involve interactors that specifically recognize them. In this case, the high sequence conservation, especially in CDS, may depend on the required conservation of that peculiar gene product rather than the presence of a G4 structure. Nonetheless, the option of targeting these conserved G4s for therapeutic purposes remains unaltered and the availability of specific and conserved loops may only enhance the chance of finding selective ligands [50]. Therefore, from this point of view, the ‘zero’ class is the best scenario for the development of specific drugs. The “negative” *loop_conservation* value scenario is of less immediate interpretation: it is possible that false positive hits fall in this category as it is unexpected that G-islands are less conserved than their connecting loops.

The evidence provided here, the previous studies on G4s in viruses, and the possibility to correctly classify the majority of viruses based on their PQSs (Table 3) suggest that most of the virus classes adopted G4-mediated mechanisms to control their viral cycles.

Together with the associated database, which is projected to be periodically updated to keep up with the fast-growing list of novel sequenced viruses, this work offers comprehensive data to guide researchers in the choice of the most significant PQSs within a human virus genome of interest. Hopefully, this will accelerate research in this area with the identification of new G4-mediated mechanisms in viruses and the development of effective and innovative therapeutics.

## Materials and methods

### PQS detection and evaluation of conservation

The complete list of viral species able to infect humans was retrieved from http://viralzone.expasy.org/all_by_species/678.html (accessed in April 2016) and, for each of them, all available complete genome sequences were downloaded from GenBank. Multiple alignments were built for each virus with usearch8 [51], using a permissive identity threshold (60%) to account for viral variability. Since in some cases nucleotide heterogeneity within viral species exceeded this value, multiple clusters of aligned sequences were obtained for some viruses, representing distinct genotypes. Considering the difficulty of obtaining high quality alignments beyond this limit of nucleotide similarity, all clusters obtained with this method were kept separate, manually assigned to specific genotypes and independently processed in the downstream analyses. One genome per each group of aligned sequences was chosen to serve as reference sequence, possibly belonging to the manually curated RefSeq database [https://www.ncbi.nlm.nih.gov/refseq/]. The complete list of selected reference sequences is reported in S1 Table.

PQSs were searched in all multiple-aligned nucleotide sequences with an in-house developed tool, as previously described [36, 52]. Briefly, a PQS was reported when at least four consecutive guanine islands (G-islands) were detected. If ‘*l’* is the minimum number of G in every G-island of a PQS and ‘*d’* is the maximum distance allowed between two consecutive G-islands, the following combinations of ‘*l’* and ‘*d’* were searched: *l* = 2 and *d* = 7; *l* = 3 and *d* = 12; *l* = 4 and *d* = 12. Patterns in the negative strands of viral genomes were searched by looking for cytosines (Cs) instead of Gs. The conservation of each PQS in the multiple aligned genomes of the viruses was determined by looking at the conservation not only of the G-islands but also their connecting loops. We computed different indexes to measure the nucleotide sequence conservation of viral genomes and PQSs:

1. *G4_scaffold_conservation_index* (*G4_SCI*): it is referred to the G-islands. For each virus and for every detected PQS, it is calculated as the percentage of independent genomes maintaining the corresponding G-islands:
  $$G4\_SCI\;=\;\frac{N_{G\_islands}}{N_{tot}}*100$$
  where N_G_islands_ is the number of sequences possessing the G-islands in a certain genome position and N_tot_ is the total number of sequences available for the virus.
2. *Loop_conservation*: it is the difference between G4_SCI and the local conservation of the viral sequence spanning the PQS (LSC_G4_).

$$Loop\_conservation\;=\;G4\_SCI-LSC_{G4}$$

LSC_G4_ is calculated as the average of LSC windows overlapping the PQS. LSC measure is computed within a sliding window of fixed length (length 20, shift 10), averaging the conservation values of each position extracted from the multiple sequence alignments with Jalview [53]. They are formally defined as:

$$LSC\;=\;\frac{\sum_{i=1}^{20}c_{\text{max}\;i}}{20}$$

$${LSC}_{G4}\;=\;\frac{{LSC}_{1}\;+\;.\;.\;.\;+{\;LSC}_{n}}{n}$$

where *c*_*max i*_ is the maximum conservation at position *i* of the multiple aligned sequences and *n* is the number of windows overlapping the PQS.The results of these analyses are presented individually for each virus and PQS (http://www.medcomp.medicina.unipd.it/main_site/doku.php?id=g4virus), together with the calculated profiles of simple linguistic complexity and Shannon entropy that can highlight other potential local features of the sequence (e.g. repeats and low complexity regions) [54]. All charts were generated with Plotly [https://plot.ly], exploiting Pandas [55] and Numpy Python libraries [56]. Multiple alignments are visualized with MSAViewer[57] and genomic features with JBrowse 1.15.0 [58]. Unless otherwise stated, analyses were conducted with ad hoc developed Python and Perl scripts, which are available in the website (scripts.tar.gz).

### Evaluation of PQS conservation in real vs randomized viral sequences

To determine whether the presence of PQSs in a virus is a conserved feature or it is only a consequence of its nucleotide composition, simulated viral genomes were generated and compared with real data. Two different strategies were adopted to generate simulated data:

1. *Single nucleotide assembling (SN)*. A computational approach was adopted where, in analogy to Huppert and Balasubramanian [43], the viral genome was modelled as a multinomial stream based on the assumption that each DNA base is independent. These authors give an explicit solution for the prevalence of PQSs in the human genome as a function of *p(G)*, the probability of any base being G. In our approach, we also accounted for the probability of cytosines (*p(C)*) and additionally assumed that adenine (A) and thymine (T) bases were equally likely to occur. As all four probabilities need to sum up to one, the statistical reference model is a multinomial distribution with probability vector *(p(G)*, *p(C)*, *p(A)*, *p(T))*. We hence took as many independent draws from this multinomial distribution as the number of nucleotides in the reference viral genome (S1 Table). The probabilities *p(G)* and *p(C)* vary for each virus and reflect the prevalence of G and C bases present in that virus, while the remaining proportion is equally split to give *p(A)* and *p(T)*. For each virus, 10,000 independent sequences were produced *in silico* with this method; the ‘sample’ R command with replacement was used and the provided parameters were the genome length and the relative abundance of the four nucleotides in the real genomes.
2. *G-islands reshuffling (ISL)*. For each virus, we generated a scaffold of length *n* made of only As, with *n* corresponding to the length of the virus reference genome; then, we replaced di-, tri-, or tetra-nucleotides, at random positions and without overlaps, with as many GG-, GGG-, GGGG-, CC-, CCC-, CCCC-islands as in the reference sequence, respectively. Overall, we generated 10,000 independent sequences for three different simulated datasets, one for each island length.

### Statistical methods

The simulated sequences were scanned for the presence of PQSs as previously described. The 10,000 counts obtained for each simulation formed the empirical distribution for PQS prevalence under the hypothesis of random assembling of the genome in the SN and ISL models respectively. The mid-P value was calculated using a homemade function. The semiparametric classifier used to assign the virus to its exact class relying on its PQS content was based on the ‘multinom’ function of the R package ‘nnet’.

### PQSs position and overlap with genomic features

The feature tables containing viral genome annotations were downloaded from RefSeq or GenBank for all the reference sequences reported in S1 Table. Genome coordinates were extracted for coding sequences (‘CDS’), repeat regions (‘repeat_region’), 5’- and 3’-untranslated (UTR) and promoter regions. Given the annotation inconsistency of promoters and UTRs, two new feature categories were created, 5’–and 3’–regulatory regions that were defined by exploiting the annotation of genes and CDSs. We calculated boundaries for genes in the positive strand of viral genomes as follows: 5’–regulatory = S_gene_ − S_cds_; 3’–regulatory = E_cds_—E_gene_. For the genes in the negative strand of viral genomes we defined: 5’–regulatory = Scds—S_gene_; 3’–regulatory = E_gene_—E_cds_. S_gene_, S_cds_, E_gene_ and E_cds_ are the Start (S) and End (E) of genes and CDSs as extracted from the feature tables. These newly defined features contain both UTRs and promoters. Since the positive genomic strands are deposited in RefSeq for most of the viruses belonging to the ssRNA (-) class, the following sequences available as negative strands were converted into their inverse complement, together with the coordinates of their genomic features: Junin arenavirus segment S (NC_005081) and segment L (NC_005080), Lassa virus segment L (NC_004297), lymphocytic choriomeningitis virus segment S (GQ862982), Machupo virus segment S (AY924208) and L (AY624354), Pichinde virus segment S (NC_006447), Rift Valley fever virus segment S (NC_014395), and Toscana virus segment S (NC_006318). The overlap extent between PQSs and genomic features was computed by intersecting the genomic coordinates of each PQS with the genomic features extracted from the corresponding virus, and a positive count was recorded every time an overlap of at least one nucleotide was detected. Finally, to compare the enrichment in different feature classes, characterized by different sizes, a normalization step was performed. The total extension of each feature class (*i*.*e*. CDS, repeat_region, 5’–regulatory and 3’–regulatory) was calculated by summing the lengths of individual features. The total count of PQS overlapping a feature class was then divided by the total length of the class and multiplied by a factor 1,000 to obtain the number of PQS present every 1,000 nucleotides. This procedure was performed considering only the PQSs conserved in at least 80% of sequences for each viral species. All feature tables files were manually revised to fix inconsistencies in the use of keywords and coordinates.

## Supporting information

S1 Fig PQS content in real vs simulated viral genomes (single nucleotide assembling). Segment diagrams of mid-P values obtained by comparing the PQS content detected in real and simulated viral genomes. Simulated viruses have the same nucleotide composition of the real ones, but different order. The three G-island types considered in the positive (+) and negative (-) strands of all human virus genomes are grouped in the 7 Baltimore classes. From left to right, each segment represents one of the three G-islands (GG, GGG, GGGG) in the positive (top half) and negative (bottom half) strands; the radius of a segment corresponds to 1 minus the mid-P value. Thus, full segments indicate highly significant PQSs, whereas null segments indicate non-significant PQSs, with respect to the random sequences.
(TIF)

S2 Fig PQS content in real vs simulated viral genomes (G-island reshuffling). Segment diagrams of mid-P values obtained by comparing the PQS content detected in real and simulated viral genomes. Simulated viruses are obtained by reshuffling the positions of their GG-, GGG- or GGGG-islands. The three G-island types considered in the positive (+) and negative (-) strands of all human virus genomes are grouped in the 7 Baltimore classes. From left to right, each segment represents one of the three G-islands (GG, GGG, GGGG) in the positive (top half) and negative (bottom half) strands; the radius of a segment corresponds to 1 minus the mid-P value. Thus, full segments indicate highly significant PQSs, whereas null segments indicate non-significant PQSs, with respect to the random sequences.
(TIF)

S3 Fig PQS overlap with genomic features—GG islands. Representative figure of GG-island PQSs that overlap with genomic features. The bar charts report the distribution of PQSs in genomic features where available and annotated in the database. The number of PQSs per 1kb is reported on the x-axis both for the positive (orange) and negative (blue) strands. The four features considered are coding sequences (CDS), repeat regions (RR), and regulatory regions at the 5’ and 3’ ends.
(TIF)

S4 Fig PQS overlap with genomic features—GGG islands. Representative figure of GGG-island PQSs that overlap with genomic features. The bar charts report the distribution of PQSs in genomic features where available and annotated in the database. The number of PQSs per 1kb is reported on the x-axis both for the positive (orange) and negative (blue) strands. The four features considered are coding sequences (CDS), repeat regions (RR), and regulatory regions at the 5’ and 3’ ends.
(TIF)

S5 Fig PQS overlap with genomic features—GGGG islands. Representative figure of GGGG-island PQSs that overlap with genomic features. The bar charts report the distribution of PQSs in genomic features where available and annotated in the database. The number of PQSs per 1kb is reported on the x-axis both for the positive (orange) and negative (blue) strands. The four features considered are coding sequences (CDS), repeat regions (RR), and regulatory regions at the 5’ and 3’ ends.
(TIF)

S1 Table Accession numbers of reference sequences selected for each virus. (DOCX)

S2 Table Experimentally validated G4s in human viruses. (DOCX)

S3 Table List of viruses whose PQS content is significant at 10% with respect to randomized sequences. (XLSX)
