# Supplementary figures and images for: G-quadruplex forming sequences in the genome of all known human viruses: A comprehensive guide
Source: PLoS Comput Biol. 2018 Dec 13;14(12):e1006675. doi: 10.1371/journal.pcbi.1006675 (PMC6307822; doi:10.1371/journal.pcbi.1006675)

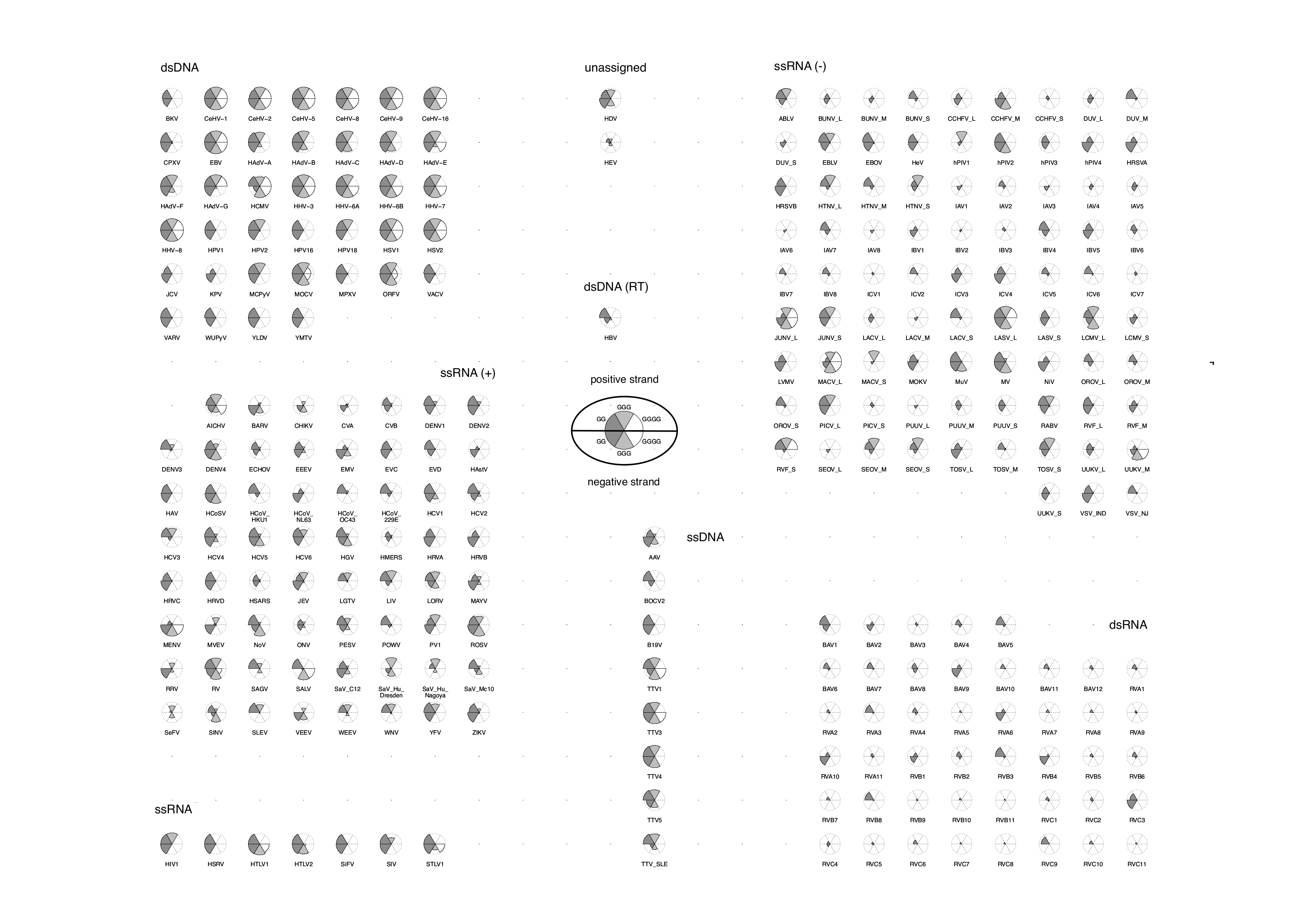

Supplement: S1 Fig — Segment diagrams of mid-P values obtained by comparing the PQS content detected in real and simulated viral genomes. Simulated viruses have the same nucleotide composition of the real ones, but different order. The three G-island types considered in the positive (+) and negative (-) strands of all human virus genomes are grouped in the 7 Baltimore classes. From left to right, each segment represents one of the three G-islands (GG, GGG, GGGG) in the positive (top half) and negative (bottom half) strands; the radius of a segment corresponds to 1 minus the mid-P value. Thus, full segments indicate highly significant PQSs, whereas null segments indicate non-significant PQSs, with respect to the random sequences. (TIF) [file pcbi.1006675.s001.tif]

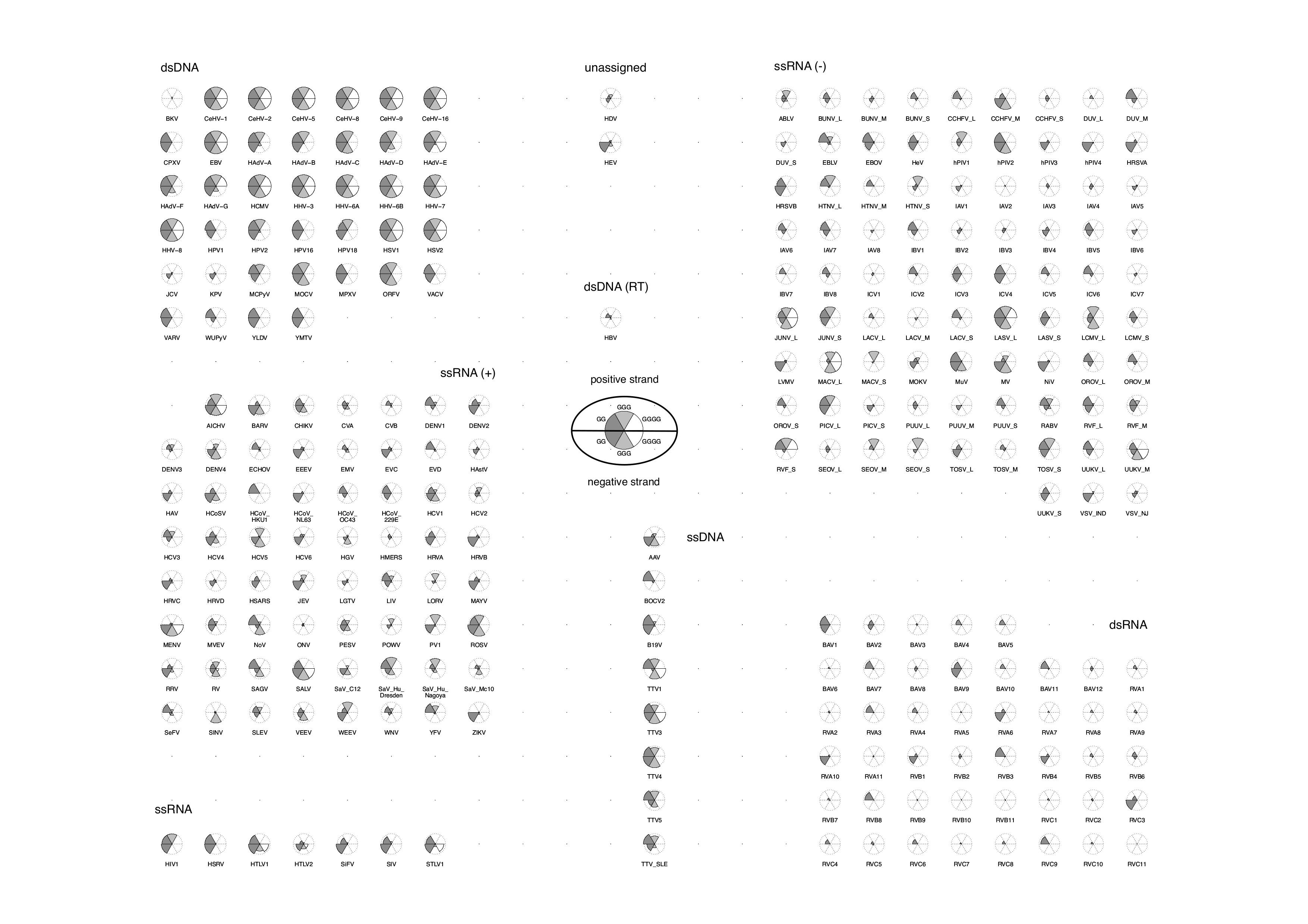

Supplement: S2 Fig — Segment diagrams of mid-P values obtained by comparing the PQS content detected in real and simulated viral genomes. Simulated viruses are obtained by reshuffling the positions of their GG-, GGG- or GGGG-islands. The three G-island types considered in the positive (+) and negative (-) strands of all human virus genomes are grouped in the 7 Baltimore classes. From left to right, each segment represents one of the three G-islands (GG, GGG, GGGG) in the positive (top half) and negative (bottom half) strands; the radius of a segment corresponds to 1 minus the mid-P value. Thus, full segments indicate highly significant PQSs, whereas null segments indicate non-significant PQSs, with respect to the random sequences. (TIF) [file pcbi.1006675.s002.tif]

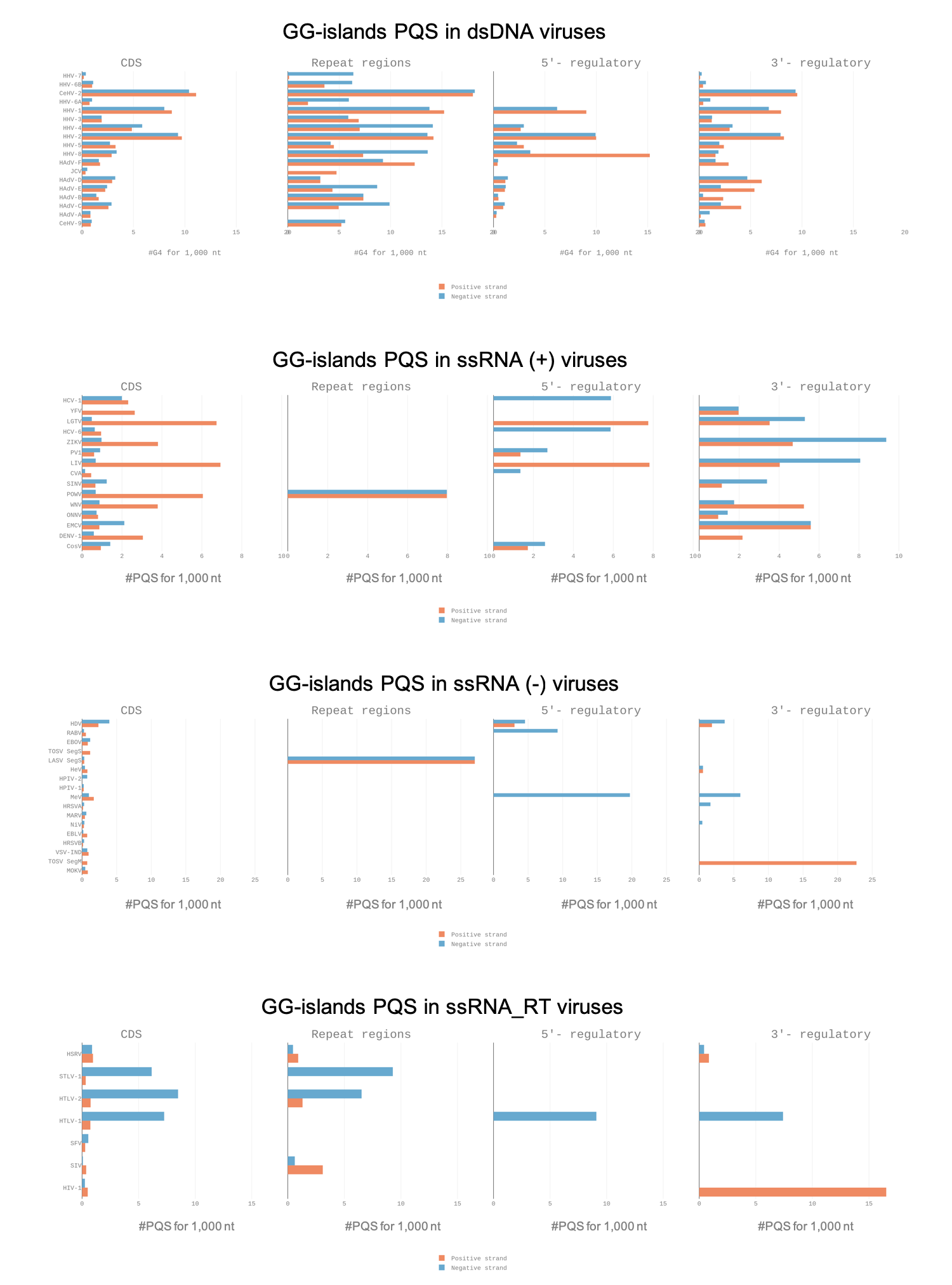

Supplement: S3 Fig — Representative figure of GG-island PQSs that overlap with genomic features. The bar charts report the distribution of PQSs in genomic features where available and annotated in the database. The number of PQSs per 1kb is reported on the x-axis both for the positive (orange) and negative (blue) strands. The four features considered are coding sequences (CDS), repeat regions (RR), and regulatory regions at the 5’ and 3’ ends. (TIF) [file pcbi.1006675.s003.tif]

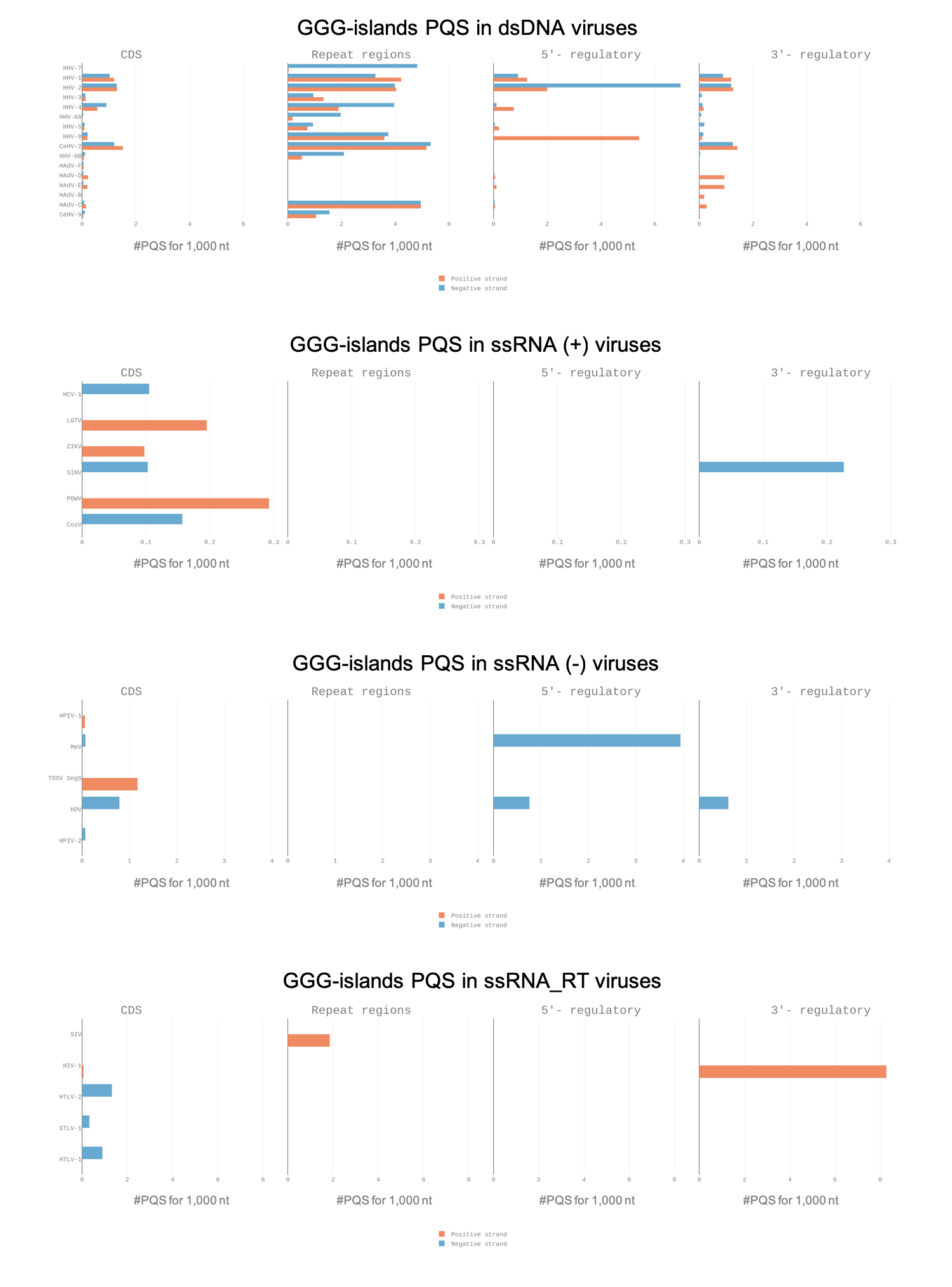

Supplement: S4 Fig — Representative figure of GGG-island PQSs that overlap with genomic features. The bar charts report the distribution of PQSs in genomic features where available and annotated in the database. The number of PQSs per 1kb is reported on the x-axis both for the positive (orange) and negative (blue) strands. The four features considered are coding sequences (CDS), repeat regions (RR), and regulatory regions at the 5’ and 3’ ends. (TIF) [file pcbi.1006675.s004.tif]

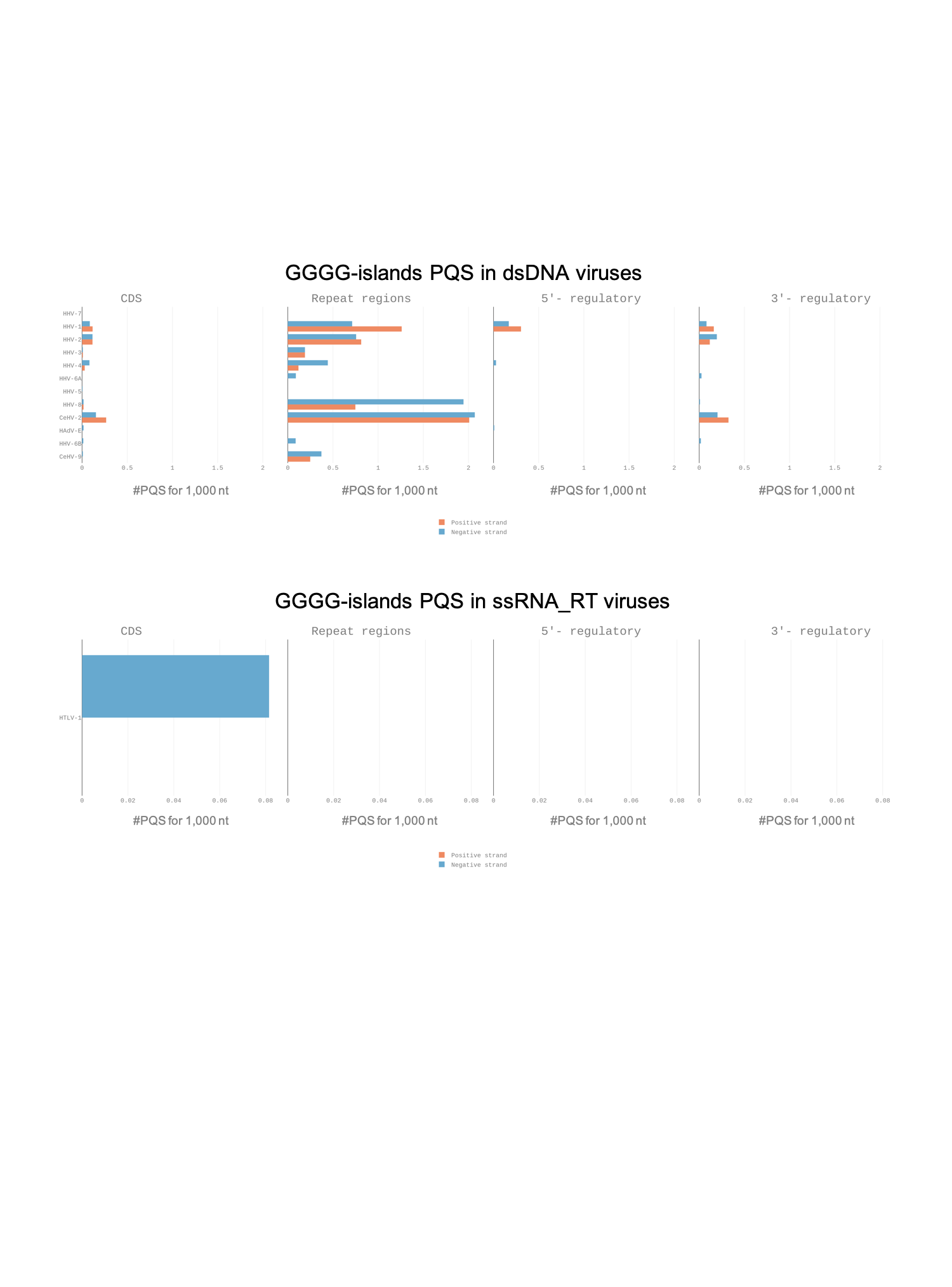

Supplement: S5 Fig — Representative figure of GGGG-island PQSs that overlap with genomic features. The bar charts report the distribution of PQSs in genomic features where available and annotated in the database. The number of PQSs per 1kb is reported on the x-axis both for the positive (orange) and negative (blue) strands. The four features considered are coding sequences (CDS), repeat regions (RR), and regulatory regions at the 5’ and 3’ ends. (TIF) [file pcbi.1006675.s005.tif]
