# Supplementary material for: G-quadruplex forming sequences in the genome of all known human viruses: A comprehensive guide
Source: PLoS Comput Biol. 2018 Dec 13;14(12):e1006675. doi: 10.1371/journal.pcbi.1006675 (PMC6307822; doi:10.1371/journal.pcbi.1006675)
Supplement: S1 Table — (DOCX) [file pcbi.1006675.s006.docx]

## Table S1. Accession numbers of reference sequences selected for each virus.

| **Virus class** | **Virus** | **Virus abbreviation** | **GenBank/RefSeq accession number** |
| --- | --- | --- | --- |
| **dsDNA (group 1)** | BK polyomavirus | BKV | NC_001538 |
|  | Cercopithecine herpesvirus type 1 | CeHV-1 | AF533768 |
|  | Cercopithecine herpesvirus type 2 | CeHV-2 | NC_006560 |
|  | Cercopithecine herpesvirus type 5 | CeHV-5 | NC_012783.2 |
|  | Cercopithecine herpesvirus type 8 | CeHV-8 | DQ120516 |
|  | Cercopithecine herpesvirus type 9 | CeHV-9 | NC_002686.2 |
|  | Cercopithecine herpesvirus type 16 | CeHV-16 | DQ149153 |
|  | Cowpox virus | CPXV | NC_003663 |
|  | Epstein Barr virus or Human herpesvirus 4 | EBV or HHV-4 | NC_007605.1 |
|  | Human adenovirus A | HAdV-A | NC_001460 |
|  | Human adenovirus B | HAdV-B | NC_011202 |
|  | Human adenovirus C | HAdV-C | NC_001405 |
|  | Human adenovirus D | HAdV-D | NC_010956 |
|  | Human adenovirus E | HAdV-E | NC_003266 |
|  | Human adenovirus F | HAdV-F | NC_001454 |
|  | Human adenovirus G | HAdV-G | DQ923122 |
|  | Human cytomegalovirus or Human herpesvirus 5 | HCMV or HHV-5 | NC_006273 |
|  | Herpes simplex virus 1 or Human herpesvirus 1 | HSV-1 or HHV-1 | NC_001806.2 |
|  | Herpes simplex virus 2 or Human herpesvirus 2 | HSV-2 or HHV-2 | NC_001798 |
|  | Human herpesvirus 6A | HHV-6A | NC_001664.2 |
|  | Human herpesvirus 6B | HHV-6B | NC_000898.1 |
|  | Human herpesvirus 7 | HHV-7 | NC_001716 |
|  | Kaposi’s sarcoma-associated herpes virus or Human herpesvirus 8 | KSHV or HHV-8 | NC_009333 |
|  | Human papillomavirus 1 | HPV 1 | NC_001356 |
|  | Human papillomavirus 2 | HPV 2 | NC_001352 |
|  | Human papillomavirus 16 | HPV 16 | K02718 |
|  | Human papillomavirus 18 | HPV 18 | NC_001357 |
|  | JC polyomavirus | JCV | NC_001699 |
|  | KI Polyomavirus | KIPyV | NC_009238 |
|  | Merkel cell polyomavirus | MCPyV | NC_010277 |
|  | Molluscum contagiosum virus | MCV | NC_001731 |
|  | Monkeypox virus | MPV | NC_003310 |
|  | Orf virus | ORFV | NC_005336 |
|  | Vaccinia virus | VACV | NC_006998 |
|  | Varicella zoster virus or Human herpesvirus 3 | VZV or HHV-3 | NC_001348 |
|  | Variola virus | VARV | NC_001611 |
|  | WU polyomavirus | WUPyV | NC_009539 |
|  | Yaba like disease virus | YLDV | NC_002642 |
|  | Yaba monkey tumor virus | YMTV | NC_005179 |
| **ssDNA (group 2)** | Adeno associated virus | AAV | NC_001401 |
|  | Human bocavirus 2 | hBoV-2 | EU082213 |
|  | Human parvovirus B19 | B19V | NC_000883 |
|  | Torque teno virus 1 | TTV-1 | NC_002076 |
|  | Torque teno virus 3 | TTV-3 | AB038620 |
|  | Torque teno virus 4 | TTV-4 | AB064602 |
|  | Torque teno virus 5 | TTV-5 | AB064606 |
|  | Torque teno virus SLE | TTV-SLE | AJ620223 |
| **dsRNA (group 3)** | Banna virus segment 1 | BAV Seg1 | NC_004211 |
|  | Banna virus segment 2 | BAV Seg2 | NC_004217 |
|  | Banna virus segment 3 | BAV3 Seg3 | KC954613 |
|  | Banna virus segment 4 | BAV4 Seg4 | NC_004219 |
|  | Banna virus segment 5 | BAV5 Seg5 | NC_004220 |
|  | Banna virus segment 6 | BAV6 Seg6 | NC_004221 |
|  | Banna virus segment 7 | BAV7 Seg7 | NC_004204 |
|  | Banna virus segment 8 | BAV8 Seg8 | NC_004203 |
|  | Banna virus segment 9 | BAV9 Seg9 | NC_004202 |
|  | Banna virus segment 10 | BAV10 Seg10 | NC_004201 |
|  | Banna virus segment 11 | BAV11 Seg11 | NC_004200 |
|  | Banna virus segment 12 | BAV12 Seg12 | NC_004198.1 |
|  | Rotavirus A segment 1 | RVA Seg1 | KU199270 |
|  | Rotavirus A segment 2 | RVA Seg2 | KU738591 |
|  | Rotavirus A segment 3 | RVA Seg3 | KU738596 |
|  | Rotavirus A segment 4 | RVA Seg4 | AB077766 |
|  | Rotavirus A segment 5 | RVA Seg5 | KU738573 |
|  | Rotavirus A segment 6 | RVA Seg6 | AB022768 |
|  | Rotavirus A segment 7 | RVA Seg7 | AB022771 |
|  | Rotavirus A segment 8 | RVA Seg8 | AB022770 |
|  | Rotavirus A segment 9 | RVA Seg9 | AB071404 |
|  | Rotavirus A segment 10 | RVA Seg10 | AB008217 |
|  | Rotavirus A segment 11 | RVA Seg11 | AB008655 |
|  | Rotavirus B segment 1 | RVB Seg1 | NC_007548 |
|  | Rotavirus B segment 2 | RVB Seg2 | NC_007549 |
|  | Rotavirus B segment 3 | RVB Seg3 | NC_007550 |
|  | Rotavirus B segment 4 | RVB Seg4 | NC_007551 |
|  | Rotavirus B segment 5 | RVB Seg5 | NC_007552 |
|  | Rotavirus B segment 6 | RVB Seg6 | NC_007553 |
|  | Rotavirus B segment 7 | RVB Seg7 | NC_007554 |
|  | Rotavirus B segment 8 | RVB Seg8 | NC_007555 |
|  | Rotavirus B segment 9 | RVB Seg9 | NC_007556 |
|  | Rotavirus B segment 10 | RVB Seg10 | NC_007557 |
|  | Rotavirus B segment 11 | RVB Seg11 | NC_007558 |
|  | Rotavirus C segment 1 | RVC Seg1 | NC_007547 |
|  | Rotavirus C segment 2 | RVC Seg2 | NC_007546 |
|  | Rotavirus C segment 3 | RVC Seg3 | NC_007572 |
|  | Rotavirus C segment 4 | RVC Seg4 | NC_007574 |
|  | Rotavirus C segment 5 | RVC Seg5 | NC_007570 |
|  | Rotavirus C segment 6 | RVC Seg6 | NC_007543 |
|  | Rotavirus C segment 7 | RVC Seg7 | NC_007544 |
|  | Rotavirus C segment 8 | RVC Seg8 | NC_007571 |
|  | Rotavirus C segment 9 | RVC Seg9 | NC_007545 |
|  | Rotavirus C segment 10 | RVC Seg10 | NC_007569 |
|  | Rotavirus C segment 11 | RVC Seg11 | NC_007573 |
| unassigned | Hepatitis E virus | HEV | NC_001434 |
| **ssRNA (+) (group 4)** | Aichi virus | AiV | NC_001918 |
|  | Barmah forest virus | BFV | NC_001786 |
|  | Chikungunya virus | CHIKV | NC_004162 |
|  | Cosavirus A | CosV | NC_012800 |
|  | Coxsackievirus A | CVA | NC_001612 |
|  | Coxsackievirus B | CVB | M16572.1 |
|  | Dengue virus 1 | DENV-1 | NC_001477 |
|  | Dengue virus 2 | DENV-2 | NC_001474.2 |
|  | Dengue virus 3 | DENV-3 | AB189128 |
|  | Dengue virus 4 | DENV-4 | AY618989 |
|  | Eastern equine encephalitis virus | EEEV | NC_003899 |
|  | Enteric Cytopathic Human Orphan virus | ECHOV | JN596587 |
|  | Encephalomyocarditis virus | EMCV | NC_001479 |
|  | Enterovirus D | EV-D | NC_001430 |
|  | GB virus C | GBV-C | NC_001710 |
|  | Hepatitis C virus genotype 1 | HCV-1 | NC_004102 |
|  | Hepatitis C virus genotype 2 | HCV-2 | NC_009823 |
|  | Hepatitis C virus genotype 3 | HCV-3 | JN588558 |
|  | Hepatitis C virus genotype 4 | HCV-4 | NC_009825 |
|  | Hepatitis C virus genotype 5 | HCV-5 | NC_009826 |
|  | Hepatitis C virus genotype 6 | HCV-6 | NC_009827 |
|  | Human astrovirus | HAstV | NC_001943 |
|  | Human coronavirus HCoV 229E | HCoV-229E | NC_002645 |
|  | Human coronavirus HCoV HKU1 | HCoV-HKU1 | NC_006577.2 |
|  | Human coronavirus HCoV NL63 | HCoV-NL63 | NC_005831 |
|  | Human coronavirus HCoV OC43 | HCoV-OC43 | KJ958218 |
|  | Enterovirus C | EV-C | AB205396 |
|  | Hepatitis A virus | HAV | K02990 |
|  | Poliovirus 1 | PV1 | NC_002058 |
|  | Rhinovirus A | RVA | NC_001617 |
|  | Rhinovirus B | RVB | NC_001490 |
|  | Rhinovirus C | RVC | EF582387 |
|  | Rhinovirus D | RVD | EF582386 |
|  | SARS coronavirus | SARS-CoV | GU553365 |
|  | Japanese encephalitis virus | JEV | NC_001437 |
|  | Langat virus | LGTV | NC_003690 |
|  | Lordsdale virus | LV | X86557 |
|  | Louping ill virus | LIV | NC_001809 |
|  | Mayaro virus | MAYV | NC_003417 |
|  | MERS coronavirus | MERS-CoV | NC_019843 |
|  | Murray valley encephalitis virus | MVEV | NC_000943 |
|  | Norovirus GI | NoV GI | NC_001959.2 |
|  | O’nyong’nyong virus | ONNV | NC_001512 |
|  | Porcine enteric sapovirus | PES | NC_000940 |
|  | Powassan virus | POWV | NC_003687 |
|  | Rosavirus 2 | RoV-2 | NC_024070 |
|  | Ross river virus | RRV | NC_001544 |
|  | Rubella virus | RuV | NC_001545 |
|  | Sagiyama virus | SAGV | AB032553 |
|  | Salivirus A | SalV-A | KP247440 |
|  | Sapovirus C12 | SaV-C12 | NC_006554 |
|  | Sapovirus hu dresden pjg sapo01 de | SaV-Hu-Dresden | NC_006269 |
|  | Sapovirus hu nagoya ngy 1 2012 jpn | SaV-Hu-Nagoya | NC_027026 |
|  | Sapovirus Mc10 | SaV-Mc10 | NC_010624 |
|  | Semliki forest virus | SeFV | NC_003215 |
|  | Sindbis virus | SINV | NC_001547 |
|  | St louis encephalitis virus | SLEV | NC_007580 |
|  | Venezuelan equine encephalitis virus | VEEV | NC_001449 |
|  | West Nile virus | WNV | NC_001563 |
|  | Western equine encephalitis virus | WEEV | KJ554965 |
|  | Yellow fever virus | YFV | NC_002031 |
|  | Zika virus | ZIKV | NC_012532 |
| unassigned | Hepatitis delta virus | HDV | NC_001653 |
| **ssRNA (-) (group 5)** | Australian bat lyssavirus | ABLV | NC_003243 |
|  | Bunyamwera virus segment L | BUNV SegL | NC_001925 |
|  | Bunyamwera virus segment M | BUNV SegM | NC_001926 |
|  | Bunyamwera virus segment S | BUNV SegS | NC_001927 |
|  | La Crosse segment L | LACV SegL | NC_004108 |
|  | La Crosse segment M | LACV SegM | NC_004109 |
|  | La Crosse segment S | LACV SegS | NC_004110 |
|  | Crimean Congo hemorrhagic fever virus segment L | CCHFV SegL | NC_005301 |
|  | Crimean Congo hemorrhagic fever virus segment M | CCHFV SegM | NC_005300 |
|  | Crimean Congo hemorrhagic fever virus segment S | CCHFV SegS | NC_005302 |
|  | Dugbe virus segment L | DUGV SegL | NC_004159 |
|  | Dugbe virus segment M | DUGV SegM | NC_004158 |
|  | Dugbe virus segment S | DUGV SegS | NC_004157 |
|  | Ebolavirus | EBOV | NC_002549 |
|  | European bat lyssavirus | EBLV | NC_009527 |
|  | Hantaan virus segment L | HTNV SegL | NC_005222 |
|  | Hantaan virus segment M | HTNV SegM | NC_005219 |
|  | Hantaan virus segment S | HTNV SegS | NC_005218 |
|  | Hendra virus | HeV | NC_001906 |
|  | Human parainfluenza virus 1 | HPIV-1 | NC_003461 |
|  | Human parainfluenza virus 2 | HPIV-2 | NC_003443 |
|  | Human parainfluenza virus 3 | HPIV-3 | NC_001796 |
|  | Human parainfluenza virus 4 | HPIV-4 | NC_021928 |
|  | Human respiratory syncytial virus A | HRSV-A | KJ672468 |
|  | Human respiratory syncytial virus B | HRSV-B | KF826843 |
|  | Influenza A virus segment 1 | IAV Seg1 | NC_002023 |
|  | Influenza A virus segment 2 | IAV Seg2 | NC_002021 |
|  | Influenza A virus segment 3 | IAV Seg3 | NC_002022 |
|  | Influenza A virus segment 4 | IAV Seg4 | NC_002017 |
|  | Influenza A virus segment 5 | IAV Seg5 | NC_002019 |
|  | Influenza A virus segment 6 | IAV Seg6 | NC_002018 |
|  | Influenza A virus segment 7 | IAV Seg7 | NC_002016 |
|  | Influenza A virus segment 8 | IAV Seg8 | NC_002020 |
|  | Influenza B virus segment 1 | IBV Seg1 | NC_002204 |
|  | Influenza B virus segment 2 | IBV Seg2 | NC_002205 |
|  | Influenza B virus segment 3 | IBV Seg3 | NC_002206 |
|  | Influenza B virus segment 4 | IBV Seg4 | NC_002207 |
|  | Influenza B virus segment 5 | IBV Seg5 | NC_002208 |
|  | Influenza B virus segment 6 | IBV Seg6 | NC_002209 |
|  | Influenza B virus segment 7 | IBV Seg7 | NC_002210 |
|  | Influenza B virus segment 8 | IBV Seg8 | NC_002211 |
|  | Influenza C virus segment 1 | ICV Seg1 | NC_006307 |
|  | Influenza C virus segment 2 | ICV Seg2 | NC_006308 |
|  | Influenza C virus segment 3 | ICV Seg3 | NC_006309 |
|  | Influenza C virus segment 4 | ICV Seg4 | NC_006310 |
|  | Influenza C virus segment 5 | ICV Seg5 | NC_006311 |
|  | Influenza C virus segment 6 | ICV Seg6 | NC_006312 |
|  | Influenza C virus segment 7 | ICV Seg7 | NC_006306 |
|  | Junin virus segment L | JUNV SegL | NC_005080 |
|  | Junin virus segment S | JUNV SegS | NC_005081 |
|  | Lake Victoria marburgvirus | MARV | NC_001608 |
|  | Lassa virus segment L | LASV SegL | NC_004297 |
|  | Lassa virus segment S | LASV SegS | NC_004296 |
|  | Lymphocytic choriomeningitis virus segment L | LCMV SegL | KJ603307 |
|  | Lymphocytic choriomeningitis virus segment S | LCMV SegS | GQ862982 |
|  | Machupo virus segment L | MACV SegL | AY624354 |
|  | Machupo virus segment S | MACV SegS | AY924208 |
|  | Measles virus | MeV | NC_001498 |
|  | Mokola virus | MOKV | NC_006429 |
|  | Mumps virus | MuV | NC_002200 |
|  | Nipah virus | NiV | NC_002728 |
|  | Oropouche virus segment L | OROV SegL | NC_005776 |
|  | Oropouche virus segment M | OROV SegM | NC_005775 |
|  | Oropouche virus segment S | OROV SegS | KP691614 |
|  | Pichinde virus segment L | PICV SegL | NC_006439 |
|  | Pichinde virus segment S | PICV SegS | NC_006447 |
|  | Puumala virus segment L | PUUV SegL | NC_005225 |
|  | Puumala virus segment M | PUUV SegM | NC_005223 |
|  | Puumala virus segment S | PUUV SegS | NC_005224 |
|  | Rabies virus | RABV | NC_001542 |
|  | Rift valley fever virus segment L | RVF SegL | NC_014397 |
|  | Rift valley fever virus segment M | RVF SegM | NC_014396 |
|  | Rift valley fever virus segment S | RVF SegS | NC_014395 |
|  | Seoul virus segment L | SEOV SegL | NC_005238 |
|  | Seoul virus segment M | SEOV SegM | NC_005237 |
|  | Seoul virus segment S | SEOV SegS | NC_005236 |
|  | Toscana virus segment L | TOSV SegL | NC_006319 |
|  | Toscana virus segment M | TOSV SegM | NC_006320 |
|  | Toscana virus segment S | TOSV SegS | NC_006318 |
|  | Uukuniemi virus segment L | UUKV SegL | NC_005214 |
|  | Uukuniemi virus segment M | UUKV SegM | NC_005220 |
|  | Uukuniemi virus segment S | UUKV SegS | KM114251 |
|  | Vesicular stomatitis virus Indiana | VSV-IND | NC_001560 |
|  | Vesicular stomatitis virus New Jersey | VSV-NJ | KU296051 |
| **ssRNA (RT) (group 6)** | Human immunodeficiency virus 1 | HIV-1 | NC_001802 |
|  | Human T-lymphotropic virus 1 | HTLV-1 | NC_001436 |
|  | Human T-lymphotropic virus 2 | HTLV-2 | AF412314 |
|  | Human spumaretrovirus | HSRV | U21247 |
|  | Simian foamy virus | SFV | NC_001364 |
|  | Simian immunodeficiency virus | SIV | NC_001549 |
|  | Simian T-lymphotropic virus 1 | STLV-1 | NC_000858 |
| **dsDNA (RT) (group 7)** | Hepatitis B virus | HBV | NC_003977 |
