# Supplementary material for: G-quadruplex forming sequences in the genome of all known human viruses: A comprehensive guide
Source: PLoS Comput Biol. 2018 Dec 13;14(12):e1006675. doi: 10.1371/journal.pcbi.1006675 (PMC6307822; doi:10.1371/journal.pcbi.1006675)
Supplement: S2 Table — (DOCX) [file pcbi.1006675.s007.docx]

## Table S2. Experimentally validated G4s in human viruses.

The table reports G4s that are detected in this analysis and previously reported in literature.

| **Reference paper** | **Virus** | **Experimentally validated G4 pattern** | **Genome coordinates†** | **G-island type** | **Genome strand** |
| --- | --- | --- | --- | --- | --- |
| ^1,2^ | HIV-1 | GGGGACTTTCCAGGGAGGCGTGGCCTGGGCGGG  GGGAGGCGTGGCCTGGGCGGGACTGGGG  GGGCGGGACTGGGGAGTGG | 9001*  9001*  9015* | GGG  GGG  GG | +  +  + |
| ^3^ | HIV-1 | GGAGGAGGAGGTGGG  GGGGGGACTGGAAGGG  CCAGTCACACCTCAGGTACCTTTAAGACC | 8527  8636  8564 | GG  GG  GG | +  +  - |
| ^4^ | HIV-1 | GGGGGGTACAGTGCAGGGG | 4347 | GG | + |
| ^5^ | HSV-1 | GGGGTTGGGGCTGGGGTTGGGG  GGGGTTGGGGTTGGGGTTGGGG  GGGGTTGGGGTTGGGGCTGGGG  GGGGCTGGGGCTGGGGCTGGGG  GGGGCTGGGGCTGGGGTTGGGG  GGGGCTGGGGTTGGGGTTGGGG  GGGGTTGGGGTTGGGGTTGGGG  GGGGGAGAGGGGAGAGGGGGGGAGAGGGG  GGGGGCGAGGGGCGGGAGGGGGCGAGGGG  GGGAGGAGCGGGGGGAGGAGCGGG | 71710*  71710*  71710*  71710*  71710*  71710*  4147  122227  9116*  117265*  151249*  126969*  202*, 152025*  126177* | GGGG  GGGG  GGGG  GGGG  GGGG  GGGG  GGGG  GGGG  GGGG  GGGG  GGGG  GGGG  GGGG  GGGG | +  +  +  +  +  +  +  -  +  -  +  -  +  - |
| ^6^ | HSV-1 | GGGAGTGGGGGTGCGTGGGAGTGGGGG  GGGGAGTGGGTGGGTGGGGAGTGGG  GGGGGCGAGGGGCGGGAGGGGGCGAGGGG  GGGGAGGGCTGGGGCCGGGGAGGGCTGGGG  CCCGCTCCTCCCCCCGCTCCTCCC  GGGTGAGGGCCGGGGGCGGGG  GGGGATTTTGGGTTGGGTCGGG | 5800*  143927*  126969*  126616*  126177*  47339  9368 | GGGG  GGGG  GGGG  GGGG  GGGG  GGG  GGG | +  +  -  -  -  +  + |
| ^6^ | HSV-2 | GGGGCGGCTGGGGCAGGGGCGGCTGGGG  GGGGGGACGGGGGGACGGGGGGACGGGGGG  GGGGGTCGGGCGGGCGGGGGTCGGG  GGGGGGCCGGGGGGACGGGGGGACGGGGGG | 72218*  133504*  153871*  148238* | GGGG  GGGG  GGGG  GGGG | +  +  +  - |
| ^7^ | EBV | GGGGCAGGAGCAGGAGGA | 96346* | GG | + |
| ^6^ | HHV-6A | GGGTTAGGGTTAGGGTTAGGG | 114*, 158062* | GGG | - |
| ^6^ | HHV-6B | GGGTTAGGGTTAGGGTTAGGG | 207*, 8485*, 153528*, 161806* | GGG | - |
| ^6^ | HHV-7 | GGGTTAGGGTTAGGGTTAGGG | 272*, 821*, 1172*, 2536*, 3658*, 9876*, 143318*, 143867*, 145582*, 146704*, 152922* | GGG | - |
| ^6^ | KSHV | GGGATGGGGGTGTGGGATGGGGG | 29998* | GGG | + |
| ^8^ | KSHV | GGGGCGGGGGACGGGGGAGGGG  GGGGCTCGGGGCTCGGGGCCCCGGGG  GGGGTCCCCCGCGGGCGGGGGCGGGG | 137468  137561  137190 | GGGG  GGGG  GGG | +  -  - |
| ^9^ | HCV-1 | GGGCTGCGGGTGGGCGGGA | 616 | GGG | + |
| ^10^ | ZIKV | GTGGAAGAGTGATAGGACTCTATGGCAATGGGGTT  GTGGAGGTGGGACGGGAG  TCGGATGTGGCAGAGGGGGCTGGAG  GCGGCGGCCGGTGTGGGGAA | 5051  7661  7905  10771 | GG  GG  GG  GG | +  +  +  + |
| ^11^ | HBV | TGGAGCTGGAGCATTCGGGCTGGG | 2986 | GG | + |
| ^9^ | EBOV | GGGGTCATATGGGAGGGATTGAAGG | 13697 | GG | + |

†Genome coordinates are referred to the whole viral genome selected as reference in this study.

* When more than four G-islands are found complying with the maximum distance allowed between consecutive G-islands, only one genome coordinate is reported corresponding to the central position of the region.

## REFERENCES

1. Perrone, R., Nadai, M., Frasson, I., et al. 2013, A dynamic G-quadruplex region regulates the HIV-1 long terminal repeat promoter, *Journal of medicinal chemistry*, 56, 6521-6530.

2. Perrone, R., Butovskaya, E., Daelemans, D., Palu, G., Pannecouque, C. and Richter, S. N. 2014, Anti-HIV-1 activity of the G-quadruplex ligand BRACO-19, *The Journal of antimicrobial chemotherapy*, 69, 3248-3258.

3. Perrone, R., Nadai, M., Poe, J. A., et al. 2013, Formation of a unique cluster of G-quadruplex structures in the HIV-1 Nef coding region: implications for antiviral activity, *PloS one*, 8, e73121.

4. Lyonnais, S., Hounsou, C., Teulade-Fichou, M. P., Jeusset, J., Le Cam, E. and Mirambeau, G. 2002, G-quartets assembly within a G-rich DNA flap. A possible event at the center of the HIV-1 genome, *Nucleic acids research*, 30, 5276-5283.

5. Artusi, S., Nadai, M., Perrone, R., et al. 2015, The Herpes Simplex Virus-1 genome contains multiple clusters of repeated G-quadruplex: Implications for the antiviral activity of a G-quadruplex ligand, *Antiviral research*, 118, 123-131.

6. Biswas, B., Kandpal, M., Jauhari, U. K. and Vivekanandan, P. 2016, Genome-wide analysis of G-quadruplexes in herpesvirus genomes, *BMC genomics*, 17, 949.

7. Murat, P., Zhong, J., Lekieffre, L., et al. 2014, G-quadruplexes regulate Epstein-Barr virus-encoded nuclear antigen 1 mRNA translation, *Nature chemical biology*, 10, 358-364.

8. Madireddy, A., Purushothaman, P., Loosbroock, C. P., Robertson, E. S., Schildkraut, C. L. and Verma, S. C. 2016, G-quadruplex-interacting compounds alter latent DNA replication and episomal persistence of KSHV, *Nucleic acids research*, 44, 3675-3694.

9. Wang, S. R., Zhang, Q. Y., Wang, J. Q., et al. 2016, Chemical Targeting of a G-Quadruplex RNA in the Ebola Virus L Gene, *Cell chemical biology*, 23, 1113-1122.

10. Fleming, A. M., Ding, Y., Alenko, A. and Burrows, C. J. 2016, Zika Virus Genomic RNA Possesses Conserved G-Quadruplexes Characteristic of the Flaviviridae Family, *ACS infectious diseases*, 2, 674-681.

11. Biswas, B., Kandpal, M. and Vivekanandan, P. 2017, A G-quadruplex motif in an envelope gene promoter regulates transcription and virion secretion in HBV genotype B, *Nucleic acids research*.
